# EphB4 inhibition defines a druggable synthetic-lethal vulnerability in MYC-driven triple-negative breast cancer

**DOI:** 10.1038/s41419-026-09020-5

**Published:** 2026-06-26

**Authors:** Zhe Sun, Yuan Zhang, Meng Ye, Zixin Wang, Zelin Pan, Xin Guo, Ziheng Zhang, Rui Wu, Ye Wu, Weidong Zhang, Xin Luan

**Affiliations:** 1 State Key Laboratory of Discovery and Utilization of Functional Components in Traditional Chinese Medicine, Shanghai Frontiers Science Center of TCM Chemical Biology, Institute of Interdisciplinary Integrative Medicine Research and Shuguang Hospital, Shanghai University of Traditional Chinese Medicine, Shanghai, China; 2 School of pharmacy, Guangdong Pharmaceutical University, Guangdong, China; 3 Department of Tissue Dynamics and Regeneration, Max Planck Institute for Multidisciplinary Sciences, Göttingen, Germany; 4 School of Pharmacy, Second Military Medical University, Shanghai, China

**Keywords:** Targeted therapies, Breast cancer

## Abstract

The MYC oncoprotein drives aggressive tumor behavior across many cancer types, yet its intrinsically disordered structure has limited direct pharmacologic targeting. Building on our previous kinome-wide CRISPR screen, we identify the receptor tyrosine kinase EphB4 as a druggable synthetic-lethal vulnerability in MYC-driven cancers. Genetic ablation or pharmacologic inhibition of EphB4 selectively triggers robust apoptosis in MYC-activated normal cells and MYC-high triple-negative breast cancer (TNBC) cell lines, while sparing MYC-low counterparts. This apoptotic response is Bcl-2-sensitive and p53-independent, overcoming a major resistance barrier in TNBC. In vivo, EphB4 inhibition markedly suppresses MYC-driven tumor growth. Notably, co-targeting EphB4 and Bcl-2 with ABT-199 yields synergistic apoptosis and induces tumor regression in TNBC models. Mechanistically, EphB4 inhibition leads to the selective transcriptional repression of PSMB5, the β5 catalytic subunit of the proteasome, resulting in the impairment of proteasome activity and the induction of MYC-dependent apoptotic stress. This establishes an unexpected link between EphB4 signaling and proteostasis maintenance, a heightened dependency in MYC-overexpressing cells due to their elevated biosynthetic load. Targeting *PSMB5* transcription, rather than its catalytic active site, also provides a potential strategy to circumvent or delay resistance to conventional proteasome inhibitors. Together, these findings define the EphB4-PSMB5 axis as a mechanistically distinct and therapeutically actionable vulnerability in MYC-high TNBC, positioning EphB4 inhibition as a promising approach to treat MYC-driven cancers.

## Introduction

MYC family oncoproteins (c-Myc, N-Myc, and L-Myc) are dysregulated in over half of human cancers, primarily through chromosomal amplification, translocation, or disruption of upstream regulatory pathways controlling MYC expression [1]. As a transcription factor, MYC regulates thousands of genes linked to core cancer hallmarks, driving intrinsic processes such as cell growth, apoptosis resistance, and metabolic reprogramming, as well as extrinsic mechanisms that remodel the tumor microenvironment and regulate antitumor immunity [2]. Studies using tetracycline-regulated MYC transgenic mouse models have further demonstrated that MYC suppression can reverse tumor progression [3–7], underscoring its essential role in tumor maintenance and suggesting that MYC inhibition could provide broad therapeutic benefit across malignancies.

Breast cancer remains a leading cause of cancer-related mortality among women, with a lifetime incidence of approximately 12-13% [8]. While tumors overexpressing HER2 or expressing estrogen and/or progesterone receptors benefit from well-established targeted therapies [9]. triple-negative breast cancer (TNBC), which lacks these receptors and comprises 15-20% of all breast cancer cases, has no widely accepted predictive biomarkers or selective therapeutic options and therefore remains a major clinical challenge [10]. TNBCs frequently exhibit elevated MYC expression and MYC-pathway dysregulation, resulting in pathway hyperactivation and poor clinical outcomes [11, 12]. Consequently, therapeutic strategies aimed at targeting MYC may hold particular promise for TNBC. However, the “undruggable” nature of MYC, stemming from its intrinsically disordered structure and lack of defined ligand-binding pockets, poses substantial challenges, and no small-molecule MYC inhibitors have yet advanced to clinical application [13, 14].

Synthetic-lethal vulnerabilities in MYC-dysregulated tumors (MYC-SL) offer an alternative strategy to indirectly target MYC-driven malignancies by exploiting dependencies on specific genes or pathways that become essential for the survival of MYC-transformed cells [15]. To uncover MYC-dependent synthetic-lethal interactions, genetic screens, such as RNAi or CRISPR-based approaches, have been performed in models where MYC is experimentally activated but not the primary transforming oncogene, including MYC-estrogen receptor fusion (MYCER)-inducible or Dox-inducible MYC expression systems [11, 16–22]. To identify novel, pharmacologically tractable targets for MYC-driven cancers, we previously conducted a kinome-wide CRISPR knockout screen in a non-transformed ARPE-19 cell model expressing a Dox-inducible MYC construct, a robust platform for discovering MYC-synthetic-lethal interactions [23, 24]. In that screen, sgRNA abundance was quantified by deep sequencing and analyzed using the MaGeCK pipeline, and candidate genes were prioritized based on negative-selection behavior in MYC-induced cells, effect size, sgRNA consistency, and downstream tractability. From this dataset, EphB4 emerged as an additional candidate MYC-associated vulnerability. EphB4, a receptor tyrosine kinase of the Eph family, regulates tumor angiogenesis, proliferation, and metastasis, making it an attractive target for cancer therapy [25, 26]. Several EphB4-targeting agents have been developed, including a soluble decoy receptor (sEphB4-HSA) that has entered clinical evaluation and selective small-molecule inhibitors such as NVP-BHG712 and AZ12672857 tested in preclinical models [27–30].

In this study, we demonstrate that EphB4 inhibition selectively induces MYC-dependent apoptosis through transcriptional repression of the proteasome subunit gene *PSMB5*, resulting in impaired proteasome activity. These findings uncover a previously unrecognized link between EphB4 signaling and proteasome regulation in the context of MYC-driven oncogenesis. Moreover, because this MYC-associated vulnerability operates independently of p53 and can be recapitulated using clinically accessible EphB4 inhibitors, our work reveals a mechanistically distinct and therapeutically actionable vulnerability in MYC-driven cancers. Given that mutations in PSMB5 frequently confer resistance to conventional proteasome inhibitors [31–33]. targeting EphB4, which suppresses PSMB5 transcription rather than its catalytic activity, may offer a viable strategy to overcome or delay such resistance. Collectively, these results establish the EphB4-PSMB5 axis as a targetable pathway and highlight EphB4 inhibition as a promising therapeutic strategy for MYC-high TNBC.

## Results

### EphB4 depletion induces MYC-dependent apoptosis

**Fig. 1:**
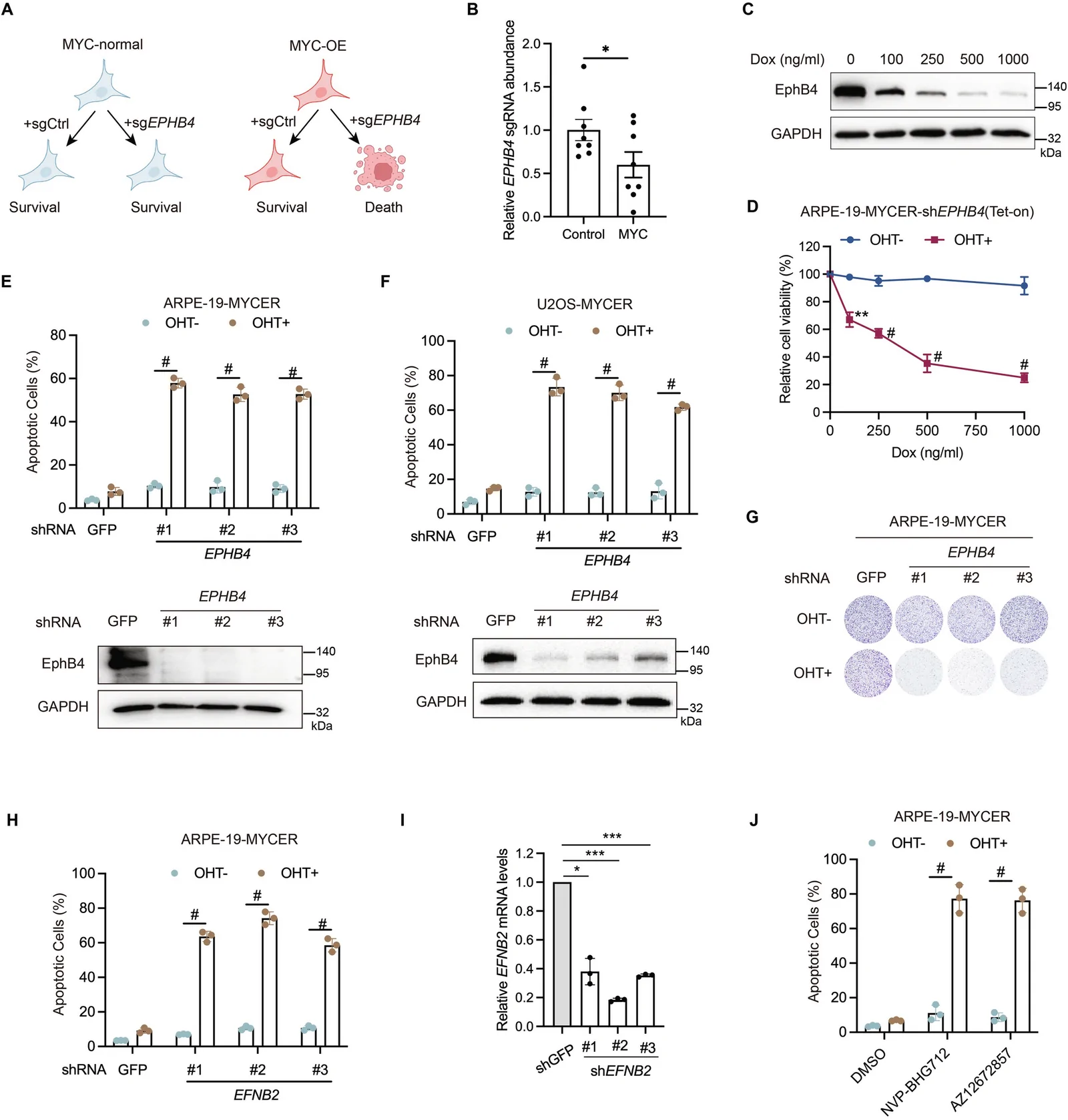
MYC-dependent vulnerability to EphB4 depletion and inhibition. **A** Schematic illustration of the screening concept showing that loss of **EPHB4** preferentially compromises the survival of MYC-overexpressing (MYC-OE) cells, but not MYC-normal cells. **B** *EPHB4* sgRNA abundances with or without MYC overexpression. **C** Immunoblot analysis showing dose-dependent suppression of EphB4 protein levels in ARPE-19-MYCER cells stably expressing doxycycline-inducible shRNAs targeting EphB4 (three independent sequences) after treatment with the indicated concentrations of doxycycline (Dox) for 48 hours. **D** Cell viability of the cells described in (**C**) following treatment with the indicated concentrations of doxycycline (Dox) for 5 days in the presence or absence of 4-hydroxytamoxifen (OHT). Apoptosis analysis of ARPE-19-MYCER (**E**) and U2OS-MYCER (**F**) cells expressing doxycycline-inducible GFP shRNA or one of three independent *EPHB4* shRNAs (#1, #2, and #3) following Dox treatment for 3 days in the presence or absence of OHT (top). Efficient EphB4 knockdown was confirmed by immunoblotting (bottom). **G** Crystal violet staining of the ARPE-19-MYCER cells described in (**E**) after Dox treatment for 5 days in the presence or absence of OHT. Apoptosis analysis of ARPE-19-MYCER cells expressing doxycycline-inducible GFP shRNA or one of three independent EFNB2 shRNAs (#1, #2, and #3), treated with Dox for 3 days in the presence or absence of OHT (**H**). Knockdown efficiency of EFNB2 was confirmed by RT-qPCR (**I**). **J** Apoptosis analysis of ARPE-19-MYCER cells treated with DMSO or the EphB4 inhibitors NVP-BHG712 (2 μM) or AZ12672857 (1 μM) for 3 days in the presence or absence of OHT. Graphical data are presented as mean ± SEM. *n* = 3–4. ^*^, *P* < 0.05; ^**^, *P* < 0.01; ^***^, *P* < 0.001; ^#^, *P* < 0.0001.

Building on our previous kinome-wide screening data, analyzed using MaGeCK, *EPHB4* emerged as a candidate gene associated with MYC-selective vulnerability (Fig. 1A). In this screen, sgRNAs targeting *EPHB4* were depleted in MYC-induced cells relative to non-induced controls (Fig. 1B), with a negative-selection score of 0.026, a log fold change of -0.55. To validate this interaction, we generated ARPE-19-MYCER cells stably expressing three independent doxycycline (Dox)-inducible shRNAs targeting *EPHB4*. Dox treatment induced a dose-dependent reduction in EphB4 protein levels (Fig. 1C; Supplementary Fig. S1A) and correspondingly decreased cell viability specifically in MYC-activated cells treated with 4-hydroxytamoxifen (OHT), with minimal effects in non-activated (OHT − ) controls (Fig. 1D). To confirm this observation, we established ARPE-19-MYCER and U2OS-MYCER cells expressing either a GFP control shRNA or one of three independent *EPHB4* shRNAs (#1, #2, or #3). Immunoblotting confirmed efficient knockdown of EphB4 by each shRNA (Fig. 1E, F, lower panels; Supplementary Fig. S1B, C). Upon MYC activation with OHT, EphB4-depleted cells exhibited marked apoptosis relative to GFP controls, and in U2OS-MYCER cells, the extent of apoptosis positively correlated with the degree of EphB4 silencing (Fig. 1E, F, upper panels). Colony-formation assays further demonstrated that EphB4 depletion profoundly suppressed clonogenic growth in MYC-activated (OHT + ) ARPE-19 cells but had negligible effects on non-activated (OHT −) counterparts (Fig. 1G).

To determine whether this MYC-selective apoptosis results from the loss of EphB4 kinase activity, we first silenced its canonical ligand, EFNB2, which potently stimulates EphB4’s intrinsic tyrosine kinase function [34]. using three independent shRNAs. EFNB2 knockdown markedly enhanced OHT-induced apoptosis, and the extent of apoptosis correlated with knockdown efficiency (Fig. 1H, I). Consistent with this, pharmacologic inhibition of EphB4 kinase activity using NVP-BHG712 or AZ12672857 selectively triggered apoptosis in OHT-treated ARPE-19-MYCER cells while sparing non-activated controls (Fig. 1J). This MYC-dependent apoptotic response was recapitulated across multiple cell types, including U2OS-MYCER, HFF-1-MYCER, and BEAS-2B-MYCER cells (Supplementary Fig. S1D–F). Together, these findings establish that MYC-activated cells are selectively vulnerable to loss of EphB4 kinase function, revealing a conserved MYC-enhanced dependency across diverse human cell contexts.

### EphB4 inhibition engages a Bcl-2-sensitive apoptotic pathway independent of p53

**Fig. 2:**
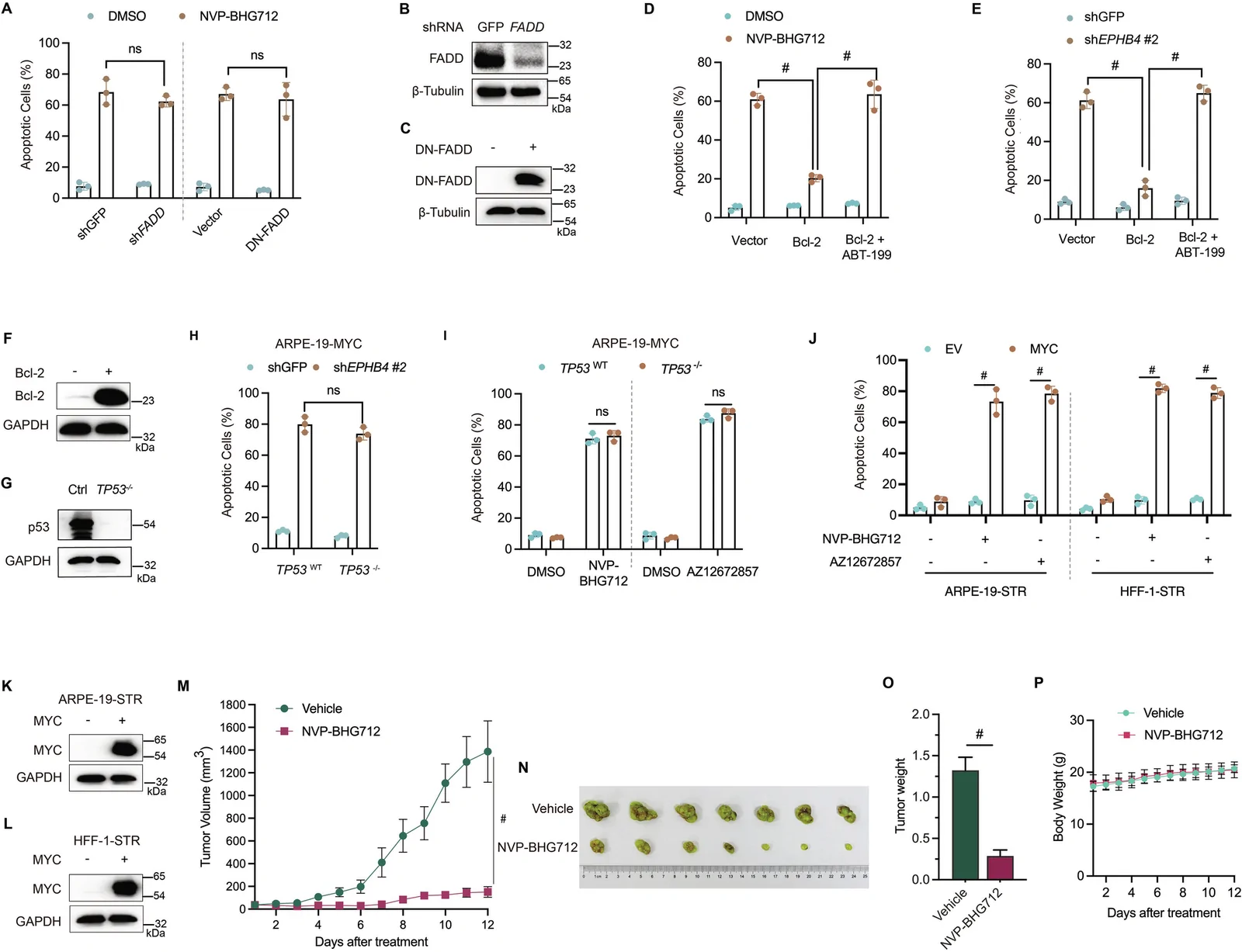
EphB4 inhibition induces Bcl-2-sensitive and p53-independent apoptosis. **A–C** ARPE-19-MYCER cells expressing GFP shRNA (control) or *FADD* shRNAs, or expressing empty vector (control) or DN-FADD, were treated with DMSO or NVP-BHG712 (2μM) for 3 days in the presence of OHT, followed by apoptosis analysis. Knockdown of FADD (**B**) and expression of DN-FADD (**C**) were confirmed by immunoblotting. **D** Apoptosis analysis of ARPE-19-MYCER cells expressing vehicle or Bcl-2 treated with OHT and the indicated compound for 3 days. NVP-BHG712, 2 μM; ABT-199, 6 μM. **E** Apoptosis analysis of ARPE-19-MYCER cells expressing either control shRNA (shGFP) or *EPHB4* shRNA (#2) in the presence of OHT. Cells were transduced with either empty vector or Bcl-2, and where indicated, treated with the Bcl-2 inhibitor ABT-199 (6 μM). **F** Immunoblotting of Bcl-2 expression in ARPE-19-MYCER cells expressing vehicle or Bcl-2. **G** Immunoblot validation of p53 knockout in ARPE-19-MYC cells. **H** Apoptosis analysis of *TP53*^WT^ and *TP53*^−/−^ ARPE-19-MYC cells expressing doxycycline-inducible GFP shRNA or *EPHB4* shRNA #2, treated with Dox for 3 days. **I** Apoptosis analysis of *TP53*^WT^ and *TP53*^−/−^ ARPE-19-MYC cells treated with DMSO, NVP-BHG712 (2 μM), or AZ12672857 (1 μM) for 3 days. **J** ARPE-19 (left) and HFF-1 (right) cells stably expressing SV40, hTERT, and HRAS^G12V^, (designated ARPE-19-STR and HFF-1-STR, respectively) were infected with a lentivirus-expressing vehicle or MYC. Subsequently, they were treated with NVP-BHG712 (2 μM) or AZ12672857 (1 μM) for 3 days, followed by apoptosis assay. MYC expression in ARPE-19-STR (**K**) and HFF-1-STR (**L**) cells was verified by immunoblotting. Nude mice bearing ARPE-19-STR-MYC xenograft tumors were treated with vehicle or 15 mg/kg NVP-BHG712 once daily intraperitoneally. The tumor volume was monitored and recorded (**M**). Tumors were excised at day 12 (**N**) and weighed (**O**). **P** The body weight of the mice was monitored throughout the 12-day treatment period. Graphical data are presented as mean ± SEM. *n* = 3–4. ns, not significant; #, *P* < 0.0001.

Apoptosis in mammalian cells is primarily executed through two core mechanisms: the death receptor-mediated extrinsic pathway and the mitochondria-mediated intrinsic pathway [35]. To delineate which pathway mediates MYC-dependent apoptosis triggered by EphB4 inhibition, we selectively blocked each cascade. The extrinsic pathway was inhibited by FADD knockdown or dominant-negative FADD (DN-FADD) overexpression, whereas the intrinsic pathway was suppressed through enforced Bcl-2 expression. FADD depletion or DN-FADD expression produced only modest effects on NVP-BHG712-induced apoptosis in OHT-treated ARPE-19-MYCER cells (Fig. 2A–C; Supplementary Fig. S2A, B), whereas Bcl-2 overexpression markedly abrogated apoptosis (Fig. 2D). Notably, co-treatment with the Bcl-2 inhibitor ABT-199 reversed this protective effect (Fig. 2D). Consistent findings were observed following shRNA-mediated *EPHB4* knockdown (Fig. 2E). Immunoblotting confirmed efficient Bcl-2 expression (Fig. 2F; Supplementary Fig. S2C). Collectively, these results demonstrate that apoptosis induced by EphB4 inhibition is mediated predominantly through the mitochondrial pathway.

Given that p53 loss-of-function mutations frequently co-occur with MYC deregulation and cooperate to drive tumor progression [36]. We next examined whether EphB4 inhibition-induced apoptosis depends on p53. Using CRISPR-Cas9, we generated p53-knockout ARPE-19-MYC cells (Fig. 2G; Supplementary Fig. S2D). Strikingly, EphB4 knockdown induced comparable levels of apoptosis in both p53 wild-type and p53-deficient cells (Fig. 2H). Likewise, pharmacologic inhibition of EphB4 with NVP-BHG712 or AZ12672857 elicited equivalent apoptotic responses across both genotypes (Fig. 2I), indicating that EphB4 inhibition induces apoptosis in MYC-activated cells independently of p53 status.

To determine whether this MYC-associated vulnerability extends to malignant contexts, we utilized previously established ARPE-19-STR and HFF-1-STR tumorigenic cell models from our laboratory [23]. These cells were rendered tumorigenic through co-expression of human telomerase reverse transcriptase (hTERT), the SV40 early region encoding large T (LT) and small T (ST) antigens, and an activated HRAS [G12V], thereby converting normal ARPE-19 and HFF-1 cells into malignant derivatives [37–39]. As expected, these transformed cells were largely resistant to treatment with NVP-BHG712 or AZ12672857 alone (Fig. 2J–L). However, ectopic MYC expression markedly sensitized them to EphB4 inhibition, resulting in apoptosis rates approaching 80% (Fig. 2J–L; Supplementary Fig. S2E, F).

This transformation-based system provides a genetically defined platform to evaluate MYC-dependent sensitivity to EphB4 inhibition in vivo. Daily intraperitoneal administration of NVP-BHG712 (15 mg/kg) markedly suppressed tumor growth, resulting in approximately 89% smaller tumor volumes and an 80% reduction in tumor weight relative to controls (Fig. 2M–O), without significant loss of body weight (Fig. 2P), indicating good tolerability in vivo. Together, these data demonstrate that EphB4 inhibition activates a Bcl-2-sensitive, p53-independent intrinsic apoptotic pathway and elicits potent, well-tolerated antitumor effects in MYC-driven malignancies.

### Triple-negative breast cancer cells with high MYC expression exhibit vulnerability to EphB4 inhibition

**Fig. 3:**
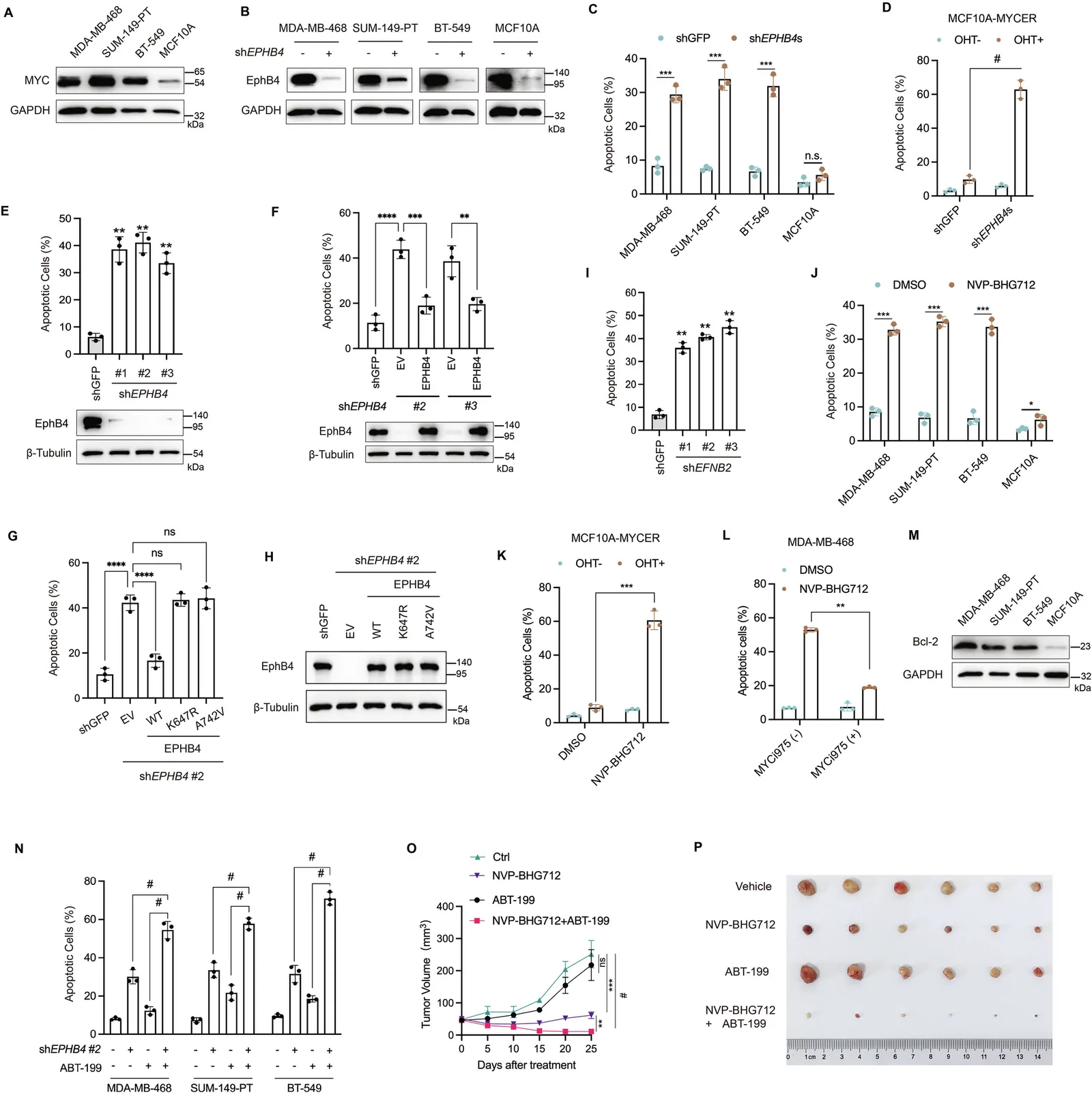
MYC-high TNBC cells are sensitive to EphB4 inhibition. **A** Immunoblotting of MYC and GAPDH in MDA-MB-468, SUM-149-PT, BT-549, and MCF10A cell lysates. The indicated cells expressing doxycycline-inducible GFP or *EPHB4* shRNAs (three independent sequences) were treated with Dox for 2 days, followed by immunoblotting of EphB4 and GAPDH (**B**), or for 5 days, followed by apoptosis assay (**C**). **D** Apoptosis analysis of MCF10A-MYCER cells expressing doxycycline-inducible GFP or *EPHB4* shRNAs (three independent sequences) treated with doxycycline for 3 days with or without OHT. **E** Apoptosis analysis of BT-549 cells expressing doxycycline-inducible GFP shRNA or one of three independent *EPHB4* shRNAs (#1, #2, or #3), treated with Dox for 3 days (top). EphB4 knockdown was confirmed by immunoblotting (bottom). Bars are mean ± SEM. *n* = 3. **, *P* < 0.01; significantly different from control cells. **F** Apoptosis analysis of *EPHB4* knockdown BT-549 cells with or without the addition of shRNA-resistant *EPHB4* cDNA (top). The expression of EphB4 was confirmed by immunoblotting (bottom). Apoptosis analysis of *EPHB4* knockdown BT-549 cells with or without the addition of shRNA-resistant *EPHB4*^*WT*^, *EPHB4*^*K647R*^, or *EPHB4*^*A742V*^ cDNA (**G**). The expression of EphB4 was confirmed by immunoblotting (**H**). **I** Apoptosis analysis of BT-549 cells stably expressing doxycycline-inducible GFP shRNA or one of three independent *EFNB2* shRNAs (#1, #2, or #3), following Dox treatment for 3 days. Bars are mean ± SEM. *n* = 3. **, *P* < 0.01; significantly different from control cells. **J** Apoptosis analysis of the indicated cells treated with DMSO or NVP-BHG712 (μM) for 3 days. **K** Apoptosis analysis of MCF10A-MYCER cells treated with DMSO or NVP-BHG712 (2 μM) with or without OHT for 3 days. **L** Apoptosis analysis of MDA-MB-468 cells treated with DMSO or NVP-BHG712 (2 μM) in the presence or absence of MYCi975 (6 μM). **M** Immunoblotting of Bcl-2 and GAPDH in MDA-MB-468, SUM-149-PT, BT-549, and MCF10A cell lysates. **N** Apoptosis analysis of MDA-MB-468, SUM-149-PT, and BT-549 cells stably expressing doxycycline-inducible *EPHB4* shRNA #2, treated with Dox (to induce *EphB4* knockdown) and/or the Bcl-2 inhibitor ABT-199 (6 μM) for 3 days. Nude mice bearing MDA-MB-468 xenograft tumors were treated with vehicle, NVP-BHG712 (15 mg/kg), and/or ABT-199 once daily intraperitoneally. The tumor volume was monitored and recorded (**O**). Tumors were excised at day 25 (**P**). Graphical data are presented as means ± SEM. *n* = 3–4. For **C, D, E, F, I, J, K, L, N**. ns, not significant; *, *P* < 0.05; **, *P* < 0.01; ***, *P* < 0.001; #, *P* < 0.0001.

Given that triple-negative breast cancers (TNBCs) frequently display elevated MYC expression yet lack effective targeted therapies, we next examined whether EphB4 inhibition selectively induces apoptosis in MYC-high TNBC cells. To assess the physiological relevance of the MYCER model and define the MYC expression status of the breast cell lines used in this study, we directly compared MYC protein levels across ARPE-19-MYCER cells and a panel of breast cell lines by immunoblotting. Under the same experimental conditions, MYCER expression in ARPE-19-MYCER cells was comparable to the endogenous MYC levels observed in the MYC-high breast cancer cell lines MDA-MB-468 and BT-549, while being markedly higher than that in the MYC-low breast cancer cell line T47D and the nonmalignant mammary epithelial cell line MCF10A (Fig. 3A; Supplementary Fig. S3A–C). These findings indicate that the MYCER model does not represent an extreme artificial overexpression setting, but rather falls within the range observed in MYC-high breast cancer cells. Based on this expression pattern, we then compared three MYC-high TNBC cell lines (MDA-MB-468, SUM-149-PT, and BT-549) with the MYC-low MCF10A cells to examine their apoptotic response to EphB4 inhibition. Co-expression of three independent *EPHB4* shRNAs efficiently reduced EphB4 protein levels in all four cell lines (Fig. 3B; Supplementary Fig. S3D); however, pronounced apoptosis occurred only in MYC-high TNBC cells, not in MCF10A, following EphB4 knockdown (Fig. 3C). Consistently, MCF10A-MYCER cells became sensitive to EphB4 depletion upon OHT treatment, exhibiting approximately 60% apoptotic cells (Fig. 3D). A similar pattern was observed in T47D-MYCER cells, in which OHT-mediated MYC activation markedly increased apoptosis induced by the EphB4 inhibitor NVP-BHG712 (Supplementary Fig. S3E). Importantly, all three MYC-high TNBC cell lines, MDA-MB-468 (*TP53*^R273H^), SUM-149-PT (*TP53*^M237I^), and BT-549 (*TP53*^R249S^), harbor missense *TP53* mutations. Despite lacking functional p53, these cells remained highly sensitive to EphB4 inhibition. This mirrors our observation that *TP53* knockout does not diminish EphB4 inhibition-induced apoptosis in ARPE-19-MYC cells, further supporting that the MYC-associated dependency on EphB4 is p53 independent.

To rule out off-target effects, we established separate cell lines, each stably expressing one of the three independent Dox-inducible *EPHB4* shRNAs (#1, #2, or #3). Dox induction markedly reduced EphB4 protein levels relative to GFP shRNA controls (Fig. 3E, bottom; Supplementary Fig. S3F) and induced a corresponding increase in apoptosis (Fig. 3E, top). To further confirm the specificity of this effect, we generated *EPHB4* cDNA constructs containing silent mutations at the shRNA #2 and #3 binding sites, rendering them resistant to the respective shRNAs. Expression of these shRNA-resistant constructs rescued cells from apoptosis induced by their corresponding shRNAs (Fig. 3F; Supplementary Fig. S3G), confirming that the observed phenotype reflects an on-target RNA interference effect.

We next asked whether this rescue depends on EphB4 kinase activity. Two kinase-dead mutations, K647R and A742V, were introduced into the shRNA #2-resistant *EPHB4* cDNA. Re-expression of wild-type EphB4 prevented apoptosis induced by EphB4 knockdown, whereas neither kinase-dead mutant conferred protection (Fig. 3G, H; Supplementary Fig. S3H), demonstrating that the kinase activity of EphB4 is essential for MYC-high TNBC cell survival. Consistently, knockdown of EFNB2 induced a comparable degree of apoptosis (Figs. 3I; S3I). Moreover, treatment with the EphB4 kinase inhibitor NVP-BHG712 triggered robust apoptosis in MYC-high TNBC cells (MDA-MB-468, SUM-149-PT, and BT-549) but not in MYC-low MCF10A cells (Fig. 3J). Strikingly, MCF10A-MYCER cells treated with OHT became sensitive to NVP-BHG712, exhibiting ~60% apoptosis (Fig. 3K). These findings demonstrate that EphB4 kinase activity is required for the survival of MYC-high TNBC cells.

To further determine whether MYC functionally contributes to the apoptotic sensitivity of TNBC cells to EphB4 inhibition, we treated MDA-MB-468 cells with MYCi975, a MYC inhibitor reported to disrupt MYC-MAX dimerization [40]. prior to exposure to NVP-BHG712. MYCi975 markedly attenuated NVP-BHG712-induced apoptosis, supporting a functional contribution of MYC to the apoptotic response triggered by EphB4 inhibition in TNBC cells (Fig. 3L). We next asked whether sensitivity to EphB4 inhibition might simply reflect high proliferative capacity rather than MYC status. Although ARPE-19-STR cells were insensitive to EphB4 inhibition, we observed that they appeared to proliferate faster than the MYC-high TNBC cell lines MDA-MB-468 and BT-549 during routine culture. We therefore formally compared their growth kinetics and MYC expression. CCK-8 and immunoblot analyses showed that ARPE-19-STR cells indeed proliferated more rapidly than MDA-MB-468 and BT-549 cells, despite expressing substantially lower levels of MYC than either TNBC line (Supplementary Fig. S3J–L). These findings argue that rapid proliferation alone is insufficient to confer sensitivity to EphB4 inhibition and instead support a closer association between EphB4 inhibitor sensitivity and MYC status.

Although both genetic and pharmacologic inhibition of EphB4 induced apoptosis in MYC-high TNBC lines, the proportion of apoptotic cells (30-40%) was lower than that observed in MYC-activated MCF10A or other MYC-activated normal cells ( ~ 60%) under identical conditions. Given that Bcl-2 overexpression markedly suppressed EphB4 inhibition-induced apoptosis in MYC-activated ARPE-19 cells (Fig. 2D, E), we hypothesized that elevated Bcl-2 expression in TNBC cells might dampen their apoptotic response. Indeed, immunoblotting revealed substantially higher Bcl-2 protein levels in MDA-MB-468, SUM-149-PT, and BT-549 cells compared with MCF10A (Fig. 3M; Supplementary Fig. S3M). To test this hypothesis, we combined Bcl-2 inhibition (ABT-199) with either EphB4 depletion or pharmacologic blockade. While ABT-199 alone induced only modest apoptosis, it markedly potentiated apoptosis triggered by EphB4 knockdown or inhibition (Fig. 3N; Supplementary Fig. S3N), indicating that dual inhibition of Bcl-2 and EphB4 synergistically enhances apoptosis in MYC-high TNBC cells.

To assess the in vivo relevance of this interaction, we treated mice bearing MDA-MB-468 xenografts with NVP-BHG712, ABT-199, or the combination. NVP-BHG712 monotherapy produced strong tumor suppression, maintaining tumors at near-baseline size throughout treatment, whereas ABT-199 alone had only a minimal effect (Fig. 3O). Strikingly, the combination regimen induced tumor regression, yielding the smallest tumor burdens at endpoint, consistent with the excised tumor masses (Fig. 3P). No significant body-weight loss was observed (Supplementary Fig. S3O), indicating good tolerability. These results demonstrate that MYC-high TNBC tumors are highly dependent on EphB4 kinase activity in vivo and that Bcl-2 co-inhibition further potentiates the antitumor response.

### PSMB5 downregulation contributes to EphB4 inhibition-induced apoptosis

**Fig. 4:**
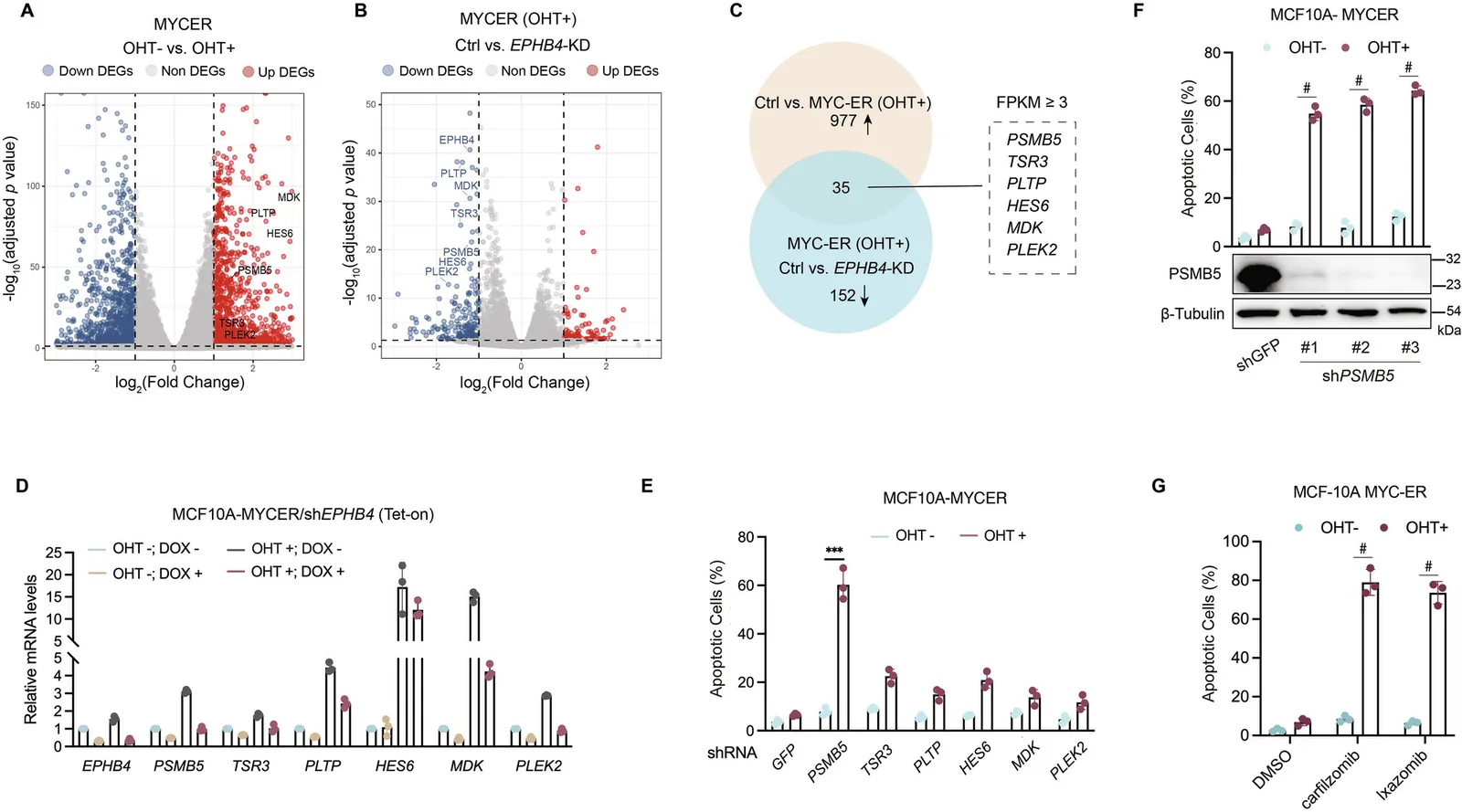
Reduction of PSMB5 is involved in EphB4 inhibition-induced apoptosis. Volcano plots of differentially expressed genes (DEGs) in MYC-activated (OHT + ) cells compared to control (OHT-) cells in MCF10A-MYCER cells (**A**), and in OHT-treated MCF10A-MYCER cells expressing GFP shRNA (Ctrl) or *EPHB4* shRNA #2 (**B**). Each dot represents one DEG, with colored circles indicating significantly upregulated or downregulated genes; *n* = 3 independent experiments. **C** Intersection of MYC-activated upregulated genes in (**A**) and EphB4 knockdown downregulated genes (**B**). For downstream validation, genes with relatively robust transcript abundance in the MYC-activated condition (fragments per kilobase of transcript per million mapped reads [FPKM] > 3) are indicated separately. **D** Relative mRNA levels of the indicated genes in MCF10A-MYCER cells expressing doxycycline-inducible *EPHB4* shRNA #2, treated with OHT and Dox alone or in combination for 3 days. **E** Apoptosis analysis of MCF10A-MYCER cells expressing three independent doxycycline-inducible shRNAs (targeting specified genes or GFP) treated with Dox for 4 days, with or without OHT. **F** Apoptosis analysis of MCF10A-MYCER cells expressing doxycycline-inducible GFP shRNA or three independent *PSMB5* shRNAs (#1, #2, or #3), treated with Dox for 3 days in the presence or absence of OHT. **G** Apoptosis analysis of MCF10A-MYCER cells treated with DMSO or the PSMB5 inhibitors ixazomib (200 nM) or carfilzomib (100 nM) for 3 days in the presence or absence of OHT. Graphical data are presented as mean ± SEM. *n* = 3–4. ***, *P* < 0.001; #, *P* < 0.0001.

To elucidate the molecular basis of the MYC-dependent apoptosis induced by EphB4 inhibition, we hypothesized that EphB4 inhibition interferes with the expression of MYC-activated survival genes, thereby selectively compromising the viability of MYC-high TNBC cells. Identifying genes that are upregulated by MYC but repressed upon EphB4 inhibition would therefore help pinpoint downstream effectors linking EphB4 signaling to MYC-driven cell survival. To this end, we performed RNA-seq analysis to identify genes positively regulated by MYC but repressed following EphB4 inhibition. Specifically, we compared the transcriptomes of MCF10A-MYCER cells with or without OHT treatment, which activates MYC, and identified 977 genes significantly upregulated upon MYC activation using a cutoff of |log2 fold change | > 1 and adjusted *p* value < 0.05 (Fig. 4A). In parallel, we analyzed gene-expression changes in OHT-treated MCF10A-MYCER cells following EphB4 knockdown and identified 152 genes significantly downregulated relative to controls using the same criteria (Fig. 4B). Intersecting these datasets revealed 35 overlapping genes. Among these overlapping genes, we further prioritized those with relatively robust transcript abundance in the MYC-activated condition (fragments per kilobase of transcript per million mapped reads [FPKM] > 3) for downstream experimental validation, yielding six candidate genes: *PSMB5, TSR3, PLTP, HES6, MDK*, and *PLEK2* (Fig. 4C; Supplementary Fig. S4A). The RNA-seq results were validated by reverse transcription-quantitative PCR (RT-qPCR) (Fig. 4D).

To assess the functional relevance of these candidates, each gene was individually silenced using a combination of three independent Dox-inducible shRNAs to ensure efficient and specific knockdown. Apoptosis was then quantified in MCF10A-MYCER cells under MYC-on and MYC-off conditions. Among the six candidates, *PSMB5* knockdown produced the most pronounced apoptotic response, resulting in approximately 60% apoptotic cells under MYC-activated conditions, whereas silencing of the other genes induced only modest apoptosis relative to GFP shRNA controls (Fig. 4E).

To rule out potential off-target effects, we generated three independent MCF10A-MYCER cell lines stably expressing individual Dox-inducible PSMB5 shRNAs (#1, #2, or #3). All three cell lines exhibited substantial reductions in PSMB5 protein levels upon Dox induction, accompanied by a significant increase in apoptosis compared with GFP shRNA controls (Fig. 4F; Supplementary Fig. S4B). Notably, the extent of apoptosis correlated with the degree of PSMB5 depletion. Consistently, pharmacologic inhibition of PSMB5 using carfilzomib or ixazomib selectively induced apoptosis in OHT-treated (MYC-activated) MCF10A-MYCER cells but not in untreated controls (Fig. 4G). Together, these findings identify PSMB5 as a key downstream effector of EphB4 inhibition and suggest that suppression of *PSMB5* transcription contributes to the apoptotic vulnerability of MYC-activated cells.

### EphB4 inhibition selectively suppresses PSMB5 transcription and impairs proteasome function in TNBC

**Fig. 5:**
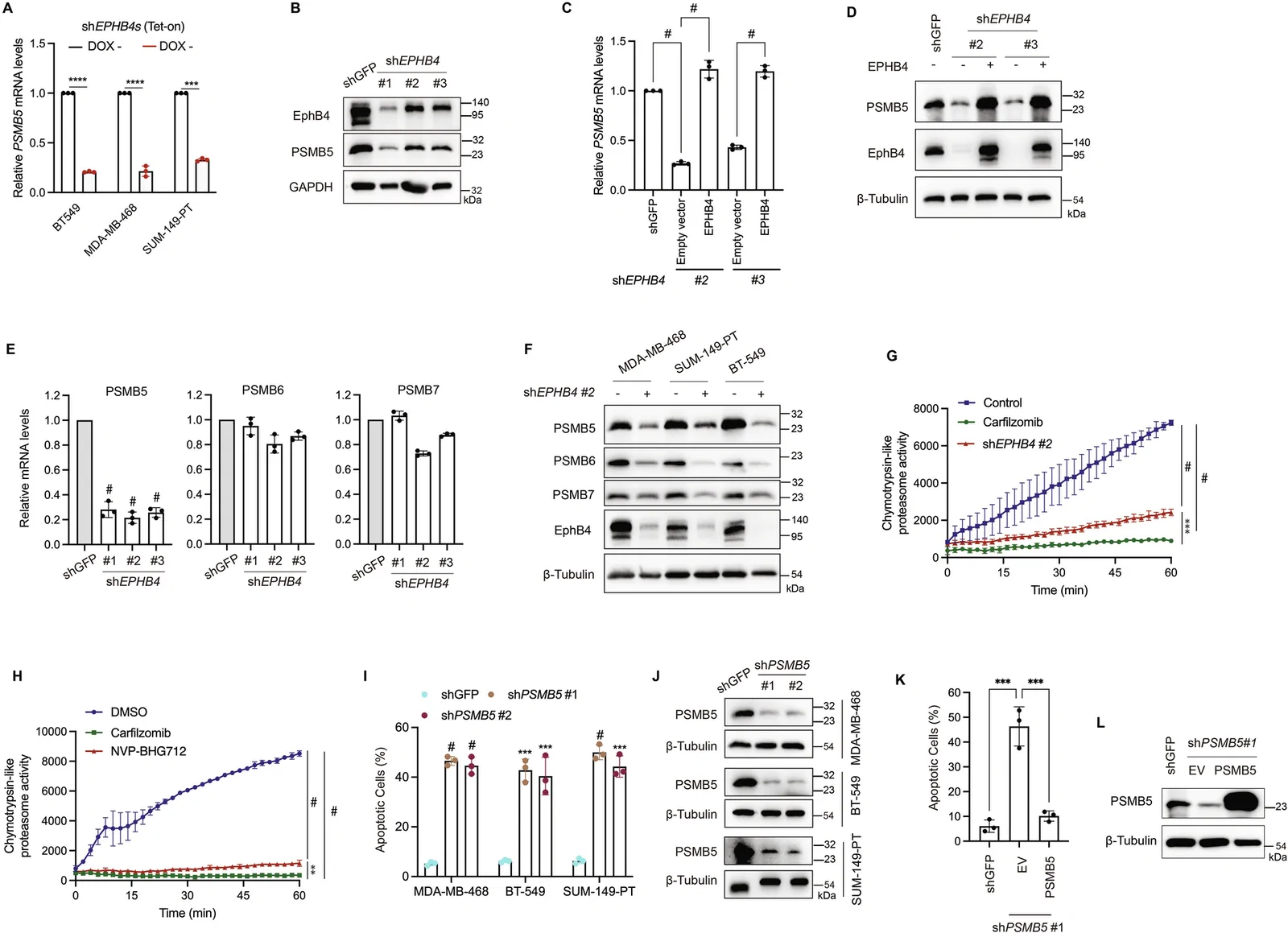
EphB4 inhibition suppresses PSMB5 transcription and impairs proteasome function in TNBC cells. **A** Relative *PSMB5* mRNA levels in indicated TNBC cells expressing doxycycline-inducible shRNAs (three independent sequences targeting *EPHB4*) and treated with Dox for 2 days. Data were normalized to β-actin and shown as mean ± SEM (*n* = 3). **B** BT-549 cells expressing Dox-inducible GFP shRNA or *EPHB4* shRNA #1, #2, or #3 were treated with Dox for 2 days. BT-549 cells expressing *EPHB4* shRNA #2 or #3, with or without the addition of shRNA-resistant *EPHB4* cDNA, were analyzed by RT-qPCR to measure *PSMB5* mRNA levels (**C**) or by immunoblotting to assess EphB4 knockdown and PSMB5 protein levels (**D**). **E** Relative *PSMB5, PSMB6*, and *PSMB7* mRNA levels in MDA-MB-468 cells expressing doxycycline-inducible GFP shRNA or three independent *EPHB4* shRNAs (#1, #2, and #3), treated with Dox for 2 days. Bars are mean ± SEM. #, *P* < 0.0001; significantly different from control cells. **F** Immunoblot analysis of the indicated proteins in MDA-MB-468, SUM-149-PT, and BT-549 cells expressing doxycycline-inducible *EPHB4* shRNA #2 after 2 days of doxycycline treatment. **G** Chymotrypsin-like proteasome activity in BT-549 cells expressing doxycycline-inducible *EphB4* shRNA #2, measured fluorometrically by Suc-LLVY-AMC peptide digestion after treatment with carfilzomib (100 nM) or doxycycline to induce EPHB4 shRNA expression for 2 days. **H** Chymotrypsin-like proteasome activity was assessed fluorometrically using the substrate Suc-LLVY-AMC in BT-549 cells treated with DMSO, carfilzomib (100 nM), or NVP-BHG712 (2 μM) for 2 days. MDA-MB-468, BT-549, and SUM-149-PT cells expressing doxycycline-inducible GFP shRNA or two independent PSMB5 shRNAs (#1, #2) were treated with Dox for 4 days followed by apoptosis analysis (**I**), or treated with Dox for 2 days and subjected to immunoblotting to verify PSMB5 knockdown (**J**). Bars are mean ± SEM. ***, *P* < 0.001; #, *P* < 0.0001; significantly different from control cells. Apoptosis analysis of *PSMB5* knockdown BT-549 cells with or without the addition of shRNA-resistant *PSMB5* cDNA (**K**). The expression of PSMB5 was confirmed by immunoblotting (**L**). For **A, C, G, H, I**, **K**. **, *P* < 0.01; ***, *P* < 0.001; #, *P* < 0.0001.

We next assessed whether the transcriptional repression of *PSMB5* by EphB4 knockdown observed in MCF10A-MYCER cells is also conserved in TNBC cells. Dox-induced silencing of *EPHB4* in BT-549, MDA-MB-468, and SUM-149-PT cells led to a marked reduction in *PSMB5* mRNA levels (Fig. 5A). Consistently, expression of any of three individual *EPHB4* shRNAs (#1, #2, or #3) decreased PSMB5 protein abundance relative to control cells, and the extent of PSMB5 reduction correlated with the efficiency of EphB4 knockdown (Fig. 5B; Supplementary Fig. S5A). In rescue experiments, overexpression of *EPHB4* cDNA constructs containing silent mutations that confer resistance to shRNA #2 or #3 effectively prevented the shRNA-induced decreases in both *PSMB5* mRNA and protein levels (Fig. 5C, D; Supplementary Fig. S5B).

Because PSMB5, PSMB6, and PSMB7 constitute the three catalytic β subunits of the 20S proteasome core, we next examined whether EphB4 inhibition also affects PSMB6 or PSMB7. Knockdown of *EPHB4* by three independent shRNAs significantly reduced *PSMB5* mRNA levels, whereas *PSMB6* and *PSMB7* transcript levels remained largely unchanged (Fig. 5E; Supplementary Fig. S5C). However, immunoblotting revealed a pronounced loss of mature PSMB6 and PSMB7 proteins concurrent with PSMB5 depletion in MDA-MB-468, SUM-149-PT, and BT-549 cells following EphB4 knockdown (Fig. 5F; Supplementary Fig. S5D), likely reflecting defective proteasome core assembly, as proper incorporation of PSMB5 is required for the maturation and stability of the other catalytic β subunits [41, 42].

We therefore asked whether these changes translate into impaired proteasome function. PSMB5, the β5 catalytic subunit of the 20S proteasome core, exhibits chymotrypsin-like activity that is essential for the degradation of ubiquitinated proteins and the maintenance of cellular proteostasis. In BT-549 cells, both genetic depletion and pharmacologic inhibition of EphB4 markedly reduced hydrolysis of the fluorogenic substrate Suc-LLVY-AMC, which reflects β5-associated chymotrypsin-like activity, to an extent comparable to that achieved with the proteasome inhibitor carfilzomib (Fig. 5G, H). A similar reduction in β5 activity was also observed in MDA-MB-468 cells upon EphB4 inhibition (Supplementary Fig. S5E). Because immunoblotting showed reduced levels of mature PSMB6 and PSMB7 proteins following EphB4 knockdown, we next examined whether their corresponding proteasome activities were also affected. Indeed, in MDA-MB-468 cells, EphB4 inhibition significantly reduced PSMB6-associated caspase-like activity and PSMB7-associated trypsin-like activity, in addition to PSMB5-associated activity (Supplementary Fig. S5E). Together, these findings provide direct evidence that EphB4 inhibition impairs proteasome function in TNBC cells.

To determine whether loss of PSMB5 is functionally linked to apoptosis in TNBC cells, we next directly targeted PSMB5. In line with results from MYC-activated MCF10A-MYCER (OHT + ) cells, PSMB5 knockdown significantly increased apoptosis in MDA-MB-468, BT-549, and SUM-149-PT cells (Fig. 5I). Efficient PSMB5 knockdown was confirmed by immunoblotting (Fig. 5J; Supplementary Fig. S5F), and re-expression of an shRNA #1-resistant *PSMB5* cDNA effectively rescued cells from shRNA #1-induced apoptosis (Fig. 5K, L; Supplementary Fig. S5G), confirming on-target specificity. Moreover, treatment of MDA-MB-468 cells with MYCi975 markedly attenuated apoptosis induced by carfilzomib (Supplementary Fig. S5H), indicating that MYC function contributes to the apoptotic vulnerability triggered by direct proteasome inhibition in this TNBC context. Collectively, these findings demonstrate that EphB4 inhibition selectively suppresses PSMB5 expression, disrupts catalytic β-subunit maturation and proteasome function, and thereby promotes MYC-dependent apoptosis in TNBC cells.

### MYC broadly activates proteasome gene transcription and enhances proteasome activity

**Fig. 6:**
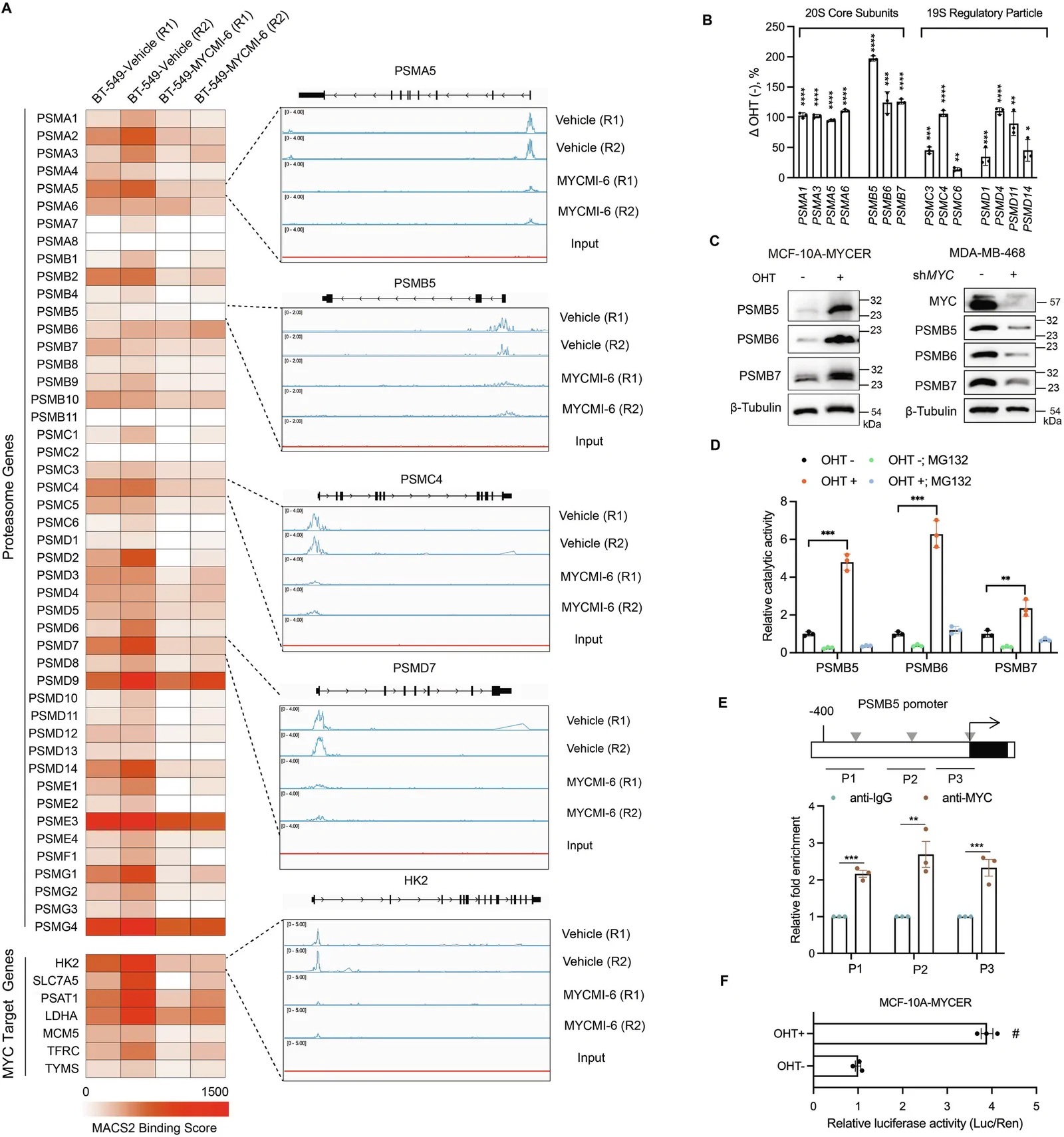
MYC activates proteasome gene transcription and enhances proteasome function. **A** Analysis of publicly available MYC ChIP-seq data (ChIP-Atlas) in BT-549 cells treated with either DMSO or MYCMI-6. **B** RT-qPCR analysis of representative proteasome subunit genes in MCF10A-MYCER cells treated with or without OHT for 2 days. Data are presented as mean ± SEM, *n* = 3. *, *P* < 0.05; **, *P* < 0.01; ***, *P* < 0.001; #, *P* < 0.0001. **C** Immunoblot analysis showing expression of the indicated proteins in MCF10A-MYCER cells treated with or without OHT for 2 days. **D** Proteasome activities associated with PSMB5, PSMB6, and PSMB7 in MCF10A-MYCER cells treated with or without OHT, as measured by fluorogenic substrate hydrolysis assays, in the absence or presence of MG132. **E** Schematic of the examined regions within the PSMB5 promoter and CUT&Tag-qPCR analysis in MDA-MB-468 cells. Three predicted MYC-binding regions (P1, P2, and P3) were analyzed using anti-MYC or control IgG antibodies. **F** Luciferase reporter assay in MCF10A-MYCER cells stably expressing a PSMB5 promoter reporter. Cells were treated with or without OHT, and luciferase activity was normalized to Renilla. For **D–F**, data are presented as mean ± SEM, *n* = 3. **, *P* < 0.01; ***, *P* < 0.001; #, *P* < 0.0001.

We hypothesized that MYC enhances proteasome gene transcription and activity to sustain proteostasis under conditions of elevated biosynthetic demand, whereas EphB4 inhibition disrupts this process by suppressing transcription of the core catalytic subunit PSMB5, leading to proteasome dysfunction and MYC-dependent apoptosis. To explore this hypothesis, we first analyzed publicly available MYC chromatin immunoprecipitation sequencing (ChIP-seq) datasets from ChIP-Atlas [43]. MYC displayed prominent binding peaks within the promoter regions of multiple proteasome subunit genes (Fig. 6A, upper panel; Supplementary Fig. S6). This enrichment was markedly reduced upon treatment with the MYC inhibitor MYCMI-6 (Fig. 6A, upper panel; Supplementary Fig. S6), which selectively disrupts the MYC-MAX interaction [44]. As a validation of this analysis, canonical MYC target genes (*HK2, SLC7A5, PSAT1, LDHA, MCM5, TFRC*, and *TYMS*) similarly exhibited strong promoter occupancy by MYC, which was abolished by MYCMI-6 (Fig. 6A, lower panel).

We next reanalyzed our previous RNA-seq data from MCF10A-MYCER cells treated with or without OHT to define how MYC regulates the proteasome. Nearly all proteasome subunit-encoding genes were upregulated upon MYC activation, with *PSMD2* being the only exception (Supplementary Fig. S7A). Targeted RT-qPCR confirmed increased expression of representative components of both the 20S core particle (*PSMA1, PSMA3, PSMA5, PSMA6, PSMB5, PSMB6*, and *PSMB7*) and the 19S regulatory complex (*PSMC3, PSMC4, PSMC6, PSMD1, PSMD4, PSMD11*, and *PSMD14*) in OHT-treated cells (Fig. 6B). At the protein level, immunoblotting revealed concordant increases in PSMB5, PSMB6, and PSMB7 in MYC-activated MCF10A-MYCER cells, whereas MYC knockdown in MDA-MB-468 cells led to pronounced reductions in these subunits (Fig. 6C; Supplementary Fig. S7B-C). Functionally, MYC activation significantly enhanced PSMB5-, PSMB6-, and PSMB7-associated proteasome activities in MCF10A-MYCER cells, as determined by fluorogenic substrate hydrolysis assays (Fig. 6D). This MYC-induced increase in proteasome activity was completely abolished by the proteasome inhibitor MG132 (Fig. 6D). These findings demonstrate that MYC directly drives the transcriptional activation of proteasome genes, resulting in coordinated upregulation of proteasome components and enhanced proteolytic capacity.

We then examined whether PSMB5 is directly regulated by MYC using targeted CUT&Tag-qPCR in MDA-MB-468 cells and luciferase reporter assays in MCF10A-MYCER cells. Three predicted MYC-binding regions (P1, P2, and P3) clustered within the proximal PSMB5 promoter region (approximately TSS − 400 to +100) showed detectable MYC enrichment by CUT&Tag-qPCR (Fig. 6E), supporting that MYC can directly engage the PSMB5 promoter (Fig. 6E). Consistently, a PSMB5 promoter-luciferase reporter spanning approximately TSS − 2000 to +100 was activated by OHT-mediated MYC induction in MCF10A-MYCER cells (Fig. 6F), further supporting that MYC promotes PSMB5 transcription through its promoter. However, inducible EPHB4 knockdown had little effect on this MYC-dependent reporter activation (Supplementary Fig. S7D), suggesting that the reduction of PSMB5 expression following EPHB4 inhibition is unlikely to be mediated primarily through altered MYC-dependent promoter transactivation.

Finally, to evaluate the clinical relevance of our findings, we analyzed publicly available breast cancer datasets. Kaplan–Meier analysis using the TCGA breast cancer cohort showed that high MYC expression and high EPHB4 expression were each associated with significantly worse 5-year overall survival (Supplementary Fig. S7E, F). Combined stratification further revealed that patients with concurrent high MYC and high EPHB4 expression exhibited poorer overall survival, whereas those with low MYC and low EPHB4 expression had the most favorable prognosis (Supplementary Fig. S7G). In addition, correlation analysis of the GEO dataset GSE25055 demonstrated a modest but significant positive correlation between MYC and EPHB4 expression, and a significant positive correlation between EPHB4 and PSMB5 expression (Supplementary Fig. S7H). Together, these data support the clinical relevance of our findings and provide clinical context for the association among MYC, EPHB4, and PSMB5 observed in this study.

## Discussion

**Fig. 7:**
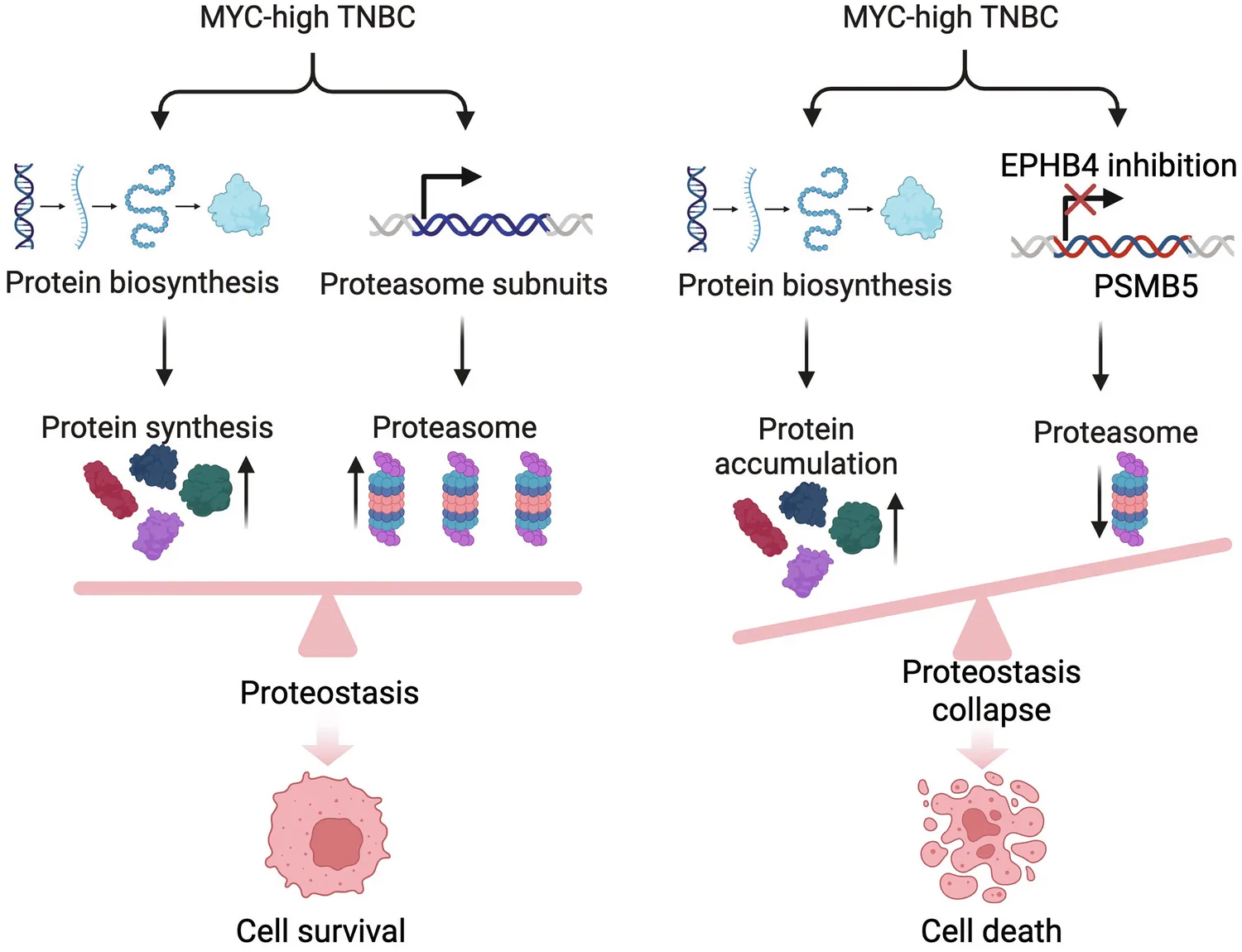
Proposed model of MYC-associated vulnerability to EphB4 inhibition. In MYC-driven cancer cells, MYC coordinately enhances protein synthesis and transcription of proteasome subunits, thereby increasing proteasome capacity and maintaining proteostasis and cell survival. Inhibition of EphB4 reduces PSMB5 expression and proteasome activity, uncoupling elevated protein synthesis from protein degradation, leading to loss of proteostasis and induction of MYC-dependent apoptosis.

MYC deregulation underlies the development and maintenance of a broad range of human malignancies, yet the lack of pharmacologically tractable binding pockets has rendered MYC itself an elusive therapeutic target. To circumvent this challenge, efforts have increasingly focused on identifying selective vulnerabilities in MYC-driven cells. Building on this premise, our study integrates genetic, pharmacologic, and transcriptomic analyses to define the receptor tyrosine kinase EphB4 as a previously unrecognized synthetic-lethal partner of MYC. We demonstrate that EphB4 inhibition, whether through genetic depletion or small-molecule blockade, represses *PSMB5* transcription, leading to impaired proteasome activity and robust MYC-dependent apoptosis. Because MYC broadly elevates transcriptional output and biosynthetic flux, MYC-driven cells exhibit heightened reliance on sustained proteasome function. Disruption of the EphB4-PSMB5 axis therefore collapses this proteostatic resilience, providing a mechanistic rationale for the observed MYC-selective cytotoxicity, as summarized in the proposed model (Fig. 7).

This MYC-associated dependency is particularly relevant for triple-negative breast cancer (TNBC), a subtype that lacks effective molecularly targeted therapies and frequently exhibits elevated MYC expression [12]. The ability of EphB4 inhibitors to phenocopy the effects of genetic depletion highlights their translational feasibility. Moreover, the selective induction of apoptosis in MYC-high TNBC cells, but not in MYC-low normal epithelial counterparts, positions MYC expression as a potential predictive biomarker for patient selection. This MYC-driven vulnerability also creates an opportunity for rational combination strategies. Consistent with the pro-apoptotic phenotype of MYC-addicted cancers, the synergistic tumor suppression observed upon dual inhibition of EphB4 and Bcl-2 suggests that pharmacologic apoptotic priming can be further leveraged. Given that inhibitors targeting both EphB4 and Bcl-2 have either reached or are progressing through clinical development, these findings offer a direct route toward clinical translation.

An additional advantage of targeting the EphB4-MYC dependency axis is its independence from functional p53. Mutations or deletions of *TP53* are highly prevalent in MYC-driven cancers, including TNBC, where they contribute to therapeutic resistance and dismal prognosis [45–47]. Our data show that EphB4 inhibition induces apoptosis irrespective of p53 status, indicating that MYC dependency, rather than intact p53 pathways, governs therapeutic response. This widens the therapeutic reach of EphB4-targeted strategies to patient populations traditionally unresponsive to p53-dependent cytotoxic agents. Furthermore, because p53-deficient tumors typically rely on compensatory stress-response mechanisms to withstand proteotoxic pressure [48–50]. disruption of the EphB4-PSMB5 axis may further intensify this vulnerability, creating a therapeutic context in which MYC-driven and p53-impaired tumors are particularly susceptible.

Mechanistically, our findings reveal a previously unexplored link between EphB4 signaling and proteasome regulation in MYC-driven cancers. The selective suppression of *PSMB5* transcription following EphB4 inhibition recapitulates the effects of direct PSMB5 blockade, leading to impaired proteasome activity and MYC-dependent apoptosis. This observation aligns with the established role of MYC in driving global transcriptional amplification, enhanced protein synthesis, and accelerated metabolic flux, processes that heighten proteotoxic stress and intensify dependence on proteasome-mediated protein clearance. By connecting a receptor tyrosine kinase to the maintenance of proteasome homeostasis, our findings broaden the conceptual landscape of MYC synthetic lethality beyond classical metabolic, replication-stress, or DNA-damage response pathways. This mechanistic insight not only deepens our understanding of MYC-driven proteostasis but also uncovers an actionable signaling-proteostasis axis.

These findings also have important implications for overcoming resistance to clinically approved proteasome inhibitors. Agents such as bortezomib, carfilzomib, and ixazomib exert potent antitumor effects by directly inhibiting the β5 catalytic activity of PSMB5 [51, 52]. However, their clinical utility has been limited by intrinsic or acquired resistance, often arising from PSMB5 mutations that impair inhibitor binding [31, 53, 54]. Targeting EphB4 may circumvent this limitation because EphB4 inhibition downregulates *PSMB5* transcription rather than engaging the catalytic site. Thus, EphB4-targeted therapy may retain efficacy against tumors harboring PSMB5 mutations that render classical proteasome inhibitors ineffective. At the same time, as with other kinase-targeted therapies, EphB4 inhibition may also be subject to intrinsic or acquired resistance, including mutational changes that impair drug binding or activation of compensatory signaling pathways. Therefore, EphB4 targeting should not be viewed as a resistance-proof strategy, but rather as a mechanistically distinct alternative that may remain effective in settings where direct PSMB5 inhibition fails. The observation that EphB4 blockade attenuates proteasome function to an extent comparable with direct PSMB5 inhibition further supports its potential role as a second-line or resistance-delaying therapeutic strategy.

While our findings establish EphB4 as a robust synthetic-lethal partner of MYC and identify PSMB5 as a critical downstream effector, several questions remain. Although CUT&Tag-qPCR and promoter-reporter assays supported that MYC can directly engage and activate the PSMB5 promoter, inducible knockdown of EPHB4 had little effect on this MYC-dependent activation, arguing against a model in which EphB4 inhibition represses PSMB5 primarily by reducing MYC occupancy or transactivation at the promoter. These findings suggest that the specific signaling mechanisms by which EphB4 inhibition represses PSMB5 expression are not yet fully defined and may involve alternative mechanisms, including MYC-independent transcriptional control, altered chromatin or co-regulator context at the PSMB5 locus, or post-transcriptional regulation of PSMB5 mRNA stability. Future studies should investigate whether canonical EphB4 downstream pathways or other regulatory mechanisms mediate this effect. In addition, although EphB4 inhibition preferentially induces apoptosis in MYC-overexpressing cells, its potential impact on proteasome function in normal tissues warrants careful evaluation to define the therapeutic window and ensure clinical safety.

In summary, our study identifies EphB4 inhibition as a mechanistically distinct, p53-independent, and pharmacologically tractable strategy for targeting MYC-driven cancers. By linking receptor tyrosine kinase signaling to proteasome regulation, we delineate a novel synthetic-lethal axis between EphB4 and MYC. These findings not only refine the conceptual framework of MYC synthetic lethality but also establish the EphB4-PSMB5 axis as a promising therapeutic target for MYC-high triple-negative breast cancer and potentially other MYC-addicted malignancies.

## Materials and methods

### Reagents and antibodies

NVP-BHG712 (S2202) and doxycycline (S5159) were purchased from Selleck Chemicals (China). ABT-199 (SJ-MX0031) was obtained from SparkJade (China). MG132 (MB5137) and carfilzomib (MB1644-1) were purchased from Meilunbio (China). AZ12672857 (T9650), ixazomib (T2122), and 4-hydroxytamoxifen (T4420) were obtained from TargetMol (China). Antibody against EphB4 (#14960) was purchased from Cell Signaling Technology (USA). Antibody against MYC (ab32072) was obtained from Abcam (USA). Antibodies against PSMB5 (19178-1-AP), p53 (10442-1-AP), Bcl-2 (12789-1-AP), FADD (14906-1-AP), and GAPDH (60004-1-Ig) were purchased from ProteinTech (China). Antibodies against PSMB6 (A4053) and PSMB7 (A14771) were obtained from ABclonal (China). The antibody against β-tubulin (M20005) was purchased from Abmart (China).

### Cell culture

ARPE-19, HFF-1, BEAS-2B, U2OS, SUM-149-PT, BT-549, and 293 T cells were maintained in Dulbecco’s Modified Eagle’s Medium (DMEM; MA0212-2, Meilunbio, China). MDA-MB-468 cells were cultured in Leibovitz’s L-15 medium (MA0547, Meilunbio, China), and BT-549 cells were cultured in Roswell Park Memorial Institute Medium 1640 (RPMI 1640; MA0215, Meilunbio, China). MCF10A cells were cultured in DMEM/F12 medium (PWL005-1, Meilunbio, China) supplemented with 10% horse serum (C2510, VivaCell, China), 20 ng/mL epidermal growth factor (EGF) (P1008, Apexbio, China), 0.5 mg/mL hydrocortisone (T1614, TargetMol, China), 5 µM forskolin (MB5959, Meilunbio, China), and 10 µg/mL insulin (T8221, TargetMol, China). U2OS-MYCER, HFF-1-MYCER, BEAS-2B-MYCER, and MCF10A-MYCER cell lines were generated by stable expression of MYC-estrogen receptor fusion (MYCER) in their respective parental cell lines. ARPE-19-STR and HFF-1-STR cells were established by stable expression of SV40 T antigen, hTERT, and oncogenic HRAS [G12V]. All cell lines, except MCF10A, were cultured in medium supplemented with 10% fetal bovine serum (C04001-500, VivaCell, China) and maintained at 37 °C in a humidified atmosphere containing 5% CO₂. All cell lines used in this study were routinely tested for mycoplasma contamination and were confirmed to be mycoplasma-free.

### Kinome-wide CRISPR-Cas9 screen and hit prioritization

The kinome-wide CRISPR-Cas9 screening data analyzed in this study were derived from our previously established screen performed in ARPE-19 cells harboring a Dox-inducible MYC construct. Briefly, a kinome-focused lentiviral sgRNA library targeting 730 kinase genes (10–14 sgRNAs per gene), together with 100 non-targeting control sgRNAs, was introduced into Cas9-expressing ARPE-19-Tet-On-MYC cells. The sgRNA vector contained a GFP reporter, and GFP-positive cells were sorted by flow cytometry to 30–50% positivity to control transduction efficiency. Sorted cells were then divided into Dox-treated and untreated groups to generate MYC-induced and control populations, respectively. After 3 days of culture, genomic DNA was isolated, sgRNA sequences were amplified, and deep sequencing was performed.

Sequencing data were analyzed using MaGeCK (v0.5.4). Candidate genes were initially evaluated based on negative-selection behavior in MYC-induced cells relative to controls, together with effect size and sgRNA consistency. For experimental follow-up, candidate prioritization also considered biological tractability and the availability of pharmacologic inhibitors. The screening metrics for EPHB4 and related candidate kinases are provided in Supplementary Table S3.

### Immunoblotting

Immunoblotting was conducted following the protocols previously outlined [55]. The presented data represent results obtained from a minimum of three independent experiments.

### ARPE-19-STR-MYC xenograft model

Female BALB/c nude mice (6 weeks old) were subcutaneously injected with 2 × 10⁶ ARPE-19-STR-MYC cells suspended in 100 μL of medium. Tumor dimensions were measured daily using a digital caliper. When the mean tumor volume reached ~50 mm³ (day 7), mice were randomly assigned (*n* = 6 per group) using a random number generator to either the vehicle or treatment arm. The vehicle group received 100 μL saline intraperitoneally each day, whereas the treatment group received NVP-BHG712 (15 mg/kg, i.p.) in a 100 μL injection. Tumor volume and body weight were monitored daily. Mice were euthanized when tumors reached 800-1500 mm³, and the excised tumors were collected and weighed. No samples or animals were excluded from the analyses. No blinding was used during group allocation, treatment administration, or outcome assessment. All manipulations involving mice were approved by the institutional animal care and use committee of Shanghai University of Traditional Chinese Medicine.

### MDA-MB-468 orthotopic xenograft model

Female BALB/c nude mice (4 weeks old) were orthotopically injected with 2 × 10⁷ MDA-MB-468 cells in 100 μL medium into the fourth mammary fat pad. Because direct cell injection showed a relatively low tumor take rate and substantial variability in tumor growth kinetics in our hands, established tumors were harvested and passaged as tumor fragments for subsequent in vivo studies to improve engraftment efficiency and growth uniformity. When tumors reached ~300 mm³, mice were euthanized and tumors were aseptically excised. Necrotic regions were removed, and viable tumor tissue was cut into 3 × 3 × 3 mm³ fragments. For implantation, the surgical site of recipient mice was sterilized, a small incision was made on the ventral side, and individual tumor fragments were implanted into the fourth mammary fat pad using a trocar. Mice were monitored daily following implantation.

Tumor dimensions were measured regularly, and once the average tumor volume reached ~50 mm³, mice were randomized into four treatment groups (*n* = 6 per group) using a random number generator:

1. Vehicle control: 100 μL intraperitoneal (i.p.) injection + 100 μL oral vehicle;
2. NVP-BHG712 monotherapy: 15 mg/kg/day, 100 μL i.p.;
3. ABT-199 monotherapy: 75 mg/kg/day, 100 μL oral gavage;
4. Combination treatment: NVP-BHG712 (15 mg/kg/day, i.p.) + ABT-199 (75 mg/kg/day, oral gavage).

Tumor volume and body weight were recorded every 5 days. Mice were sacrificed when tumors reached 300-500 mm³, and tumors were collected and weighed. No samples or animals were excluded from the analyses. No blinding was used during group allocation, treatment administration, or outcome assessment. All manipulations involving mice were approved by the institutional animal care and use committee of Shanghai University of Traditional Chinese Medicine.

### Lentiviral shRNA cloning, production, and infection

shRNA oligonucleotides were annealed and ligated into the pLKO-Tet-On vector at the AgeI and EcoRI restriction sites downstream of the H1/TO promoter. The shRNA sequences are listed in Table S1. Lentiviral particles were generated by co-transfecting 293 T cells with the shRNA vector, psPAX2, and pMD2.G packaging plasmids. Viral supernatants were collected, passed through a 0.45-µm PES filter (Jet Biofil, China), and used to infect target cells.

### RNA extraction and quantitative real-time PCR

Total RNA was isolated as previously described [55]. cDNA was synthesized using the 1st Strand cDNA Synthesis SuperMix for qPCR (Yeasen, China). qRT-qPCR was performed on an ABI 7500 system, and gene expression levels were normalized to *ACTB* (β-actin). Primer sequences are listed in Table S2.

### RNA sequencing and differential expression analysis

Total RNA was isolated using TRIzol reagent from MCF10A-MYCER cells treated with or without OHT, and from OHT-treated MCF10A-MYCER cells expressing doxycycline-inducible *EPHB4* shRNA #2 with or without doxycycline treatment. RNA sequencing and bioinformatic analysis were performed by Azenta Life Sciences (Shanghai, China). Raw sequencing data were generated using Bcl2fastq (v2.20.0.422). Sequence quality was assessed using FastQC (v0.10.1), and reads were filtered using Cutadapt (v1.9.1) by removing adapter sequences, trimming low-quality bases (quality score <20) and reads containing N bases at the 5′ or 3′ ends, and excluding reads shorter than 75 bp after trimming. Clean reads were aligned to the human reference genome (hg38) using HISAT2 (v2.2.1) with default parameters. Gene-level read counts were used as input for differential expression analysis, which was performed using DESeq2 (v1.26.0). Genes with |log2 fold change | > 1 and adjusted *p* value < 0.05 were considered differentially expressed. Replicate consistency was evaluated by correlation analysis and principal component analysis (PCA). Among the overlapping genes identified from the MYC-upregulated gene set and the genes downregulated following EphB4 knockdown, transcripts with relatively robust abundance in the MYC-activated condition (FPKM > 3) were further prioritized for downstream experimental validation; this threshold was used for candidate prioritization only and not for DEG calling. The RNA sequencing result are provided in Supplementary Table S4.

### Cell viability and apoptosis assay

Cell viability was measured using the Cell Counting Kit-8 (K1018, APExBIO, China) according to the manufacturer’s protocol. Apoptosis was assessed by Annexin V/PI staining. Pacific Blue™ Annexin V (BioLegend, 640918), Annexin V–FITC and Annexin V–Alexa Fluor 647 (Yeasen, 40302ES60 and 40304ES60), and Propidium Iodide (Beyotime, ST511) were used as indicated. Stained cells were analyzed on a BD LSRFortessa X-20 flow cytometer, and data were processed using FlowJo v10.6.

### PSMB5 promoter reporter assay

A lentiviral firefly luciferase reporter construct containing the human *PSMB5* promoter region ( − 2000 to +100 relative to the transcription start site) was generated. Lentiviral particles were produced in 293 T cells and used to infect the indicated cells to establish stable *PSMB5* promoter reporter cell lines. A Renilla luciferase construct was used as an internal control. Cells were harvested and lysed, and luciferase activities were measured using a Dual-Luciferase Reporter Assay Kit (Meilunbio) according to the manufacturer’s instructions. Firefly luciferase activity was normalized to Renilla luciferase activity, and relative luciferase activity was expressed as the firefly/Renilla ratio (Luc/Ren).

### CUT&Tag–qPCR assay

CUT&Tag–qPCR was performed to assess MYC occupancy at the *PSMB5* promoter using the Hyperactive Universal CUT&Tag Assay Kit for qPCR (Vazyme, HD103) according to the manufacturer’s instructions. Briefly, cells (5 × 10⁵ per sample) were collected and immobilized on concanavalin A–coated magnetic beads, followed by incubation with primary antibody overnight at 4°C. An anti-MYC antibody was used for target detection, and normal IgG served as a negative control. After antibody incubation, samples were treated with protein A/G–MNase fusion protein, followed by targeted chromatin fragmentation. The reaction was activated by CaCl₂ and terminated by addition of stop buffer. Released DNA fragments were purified using magnetic bead–based extraction. Enriched DNA was analyzed by quantitative PCR using primers targeting three predicted MYC-binding regions (P1, P2, and P3) clustered within the proximal *PSMB5* promoter region (approximately TSS − 400 to +100). The primer sequences were as follows: P1-F, 5′-CACTGAGACTCCCTGGACCT-3′; P1-R, 5′-CACACTAGACATGGCGCTTG-3′; P2-F, 5′-GCCGACCTCACTTCCCTTAC-3′; P2-R, 5′-TTTTCACTGAAGGGGGTCGG-3′; P3-F, 5′-TCTATTTCCCCGACCCCCTT-3′; and P3-R, 5′-CCGTCACAGCTTCACTTCCT-3′. Relative enrichment was calculated using the 2^−ΔΔCt method with spike-in DNA as an internal reference and normalized to the IgG control.

### Proteasome activity assay

Cells were treated as indicated and lysed in ice-cold proteasome lysis buffer (25 mM HEPES, pH 7.2; 50 mM NaCl; 5 mM MgCl₂; 2 mM ATP; 1 mM DTT; 10% glycerol; and 0.2% Triton X-100). Lysates were incubated on ice for 30 min with gentle pipetting every 5 min, followed by centrifugation at 12,000 × *g* for 15 min at 4°C. The supernatant was collected, and protein concentrations were determined using a BCA Protein Assay Kit. For proteasome activity measurements, 10 μg of total protein was mixed with 90 μL of reaction buffer (composition identical to the lysis buffer but lacking Triton X-100) and supplemented with the fluorogenic substrates Suc-LLVY-AMC (K2242, APExBIO), Z-LLE-AMC (HY-123053, MCE), or Boc-LRR-AMC TFA (SJ-BP0293, SparkJade) at a final concentration of 100 μM to assess chymotrypsin-like (β5), caspase-like (β1), and trypsin-like (β2) activities, respectively. The reaction mixtures were transferred to a black 96-well plate, and AMC fluorescence was recorded at 37°C using a microplate reader (excitation, 355 nm; emission, 460 nm) at 1-min intervals for 60 min.

### Public dataset-based survival and correlation analyses

To assess the clinical relevance of our findings, public breast cancer datasets were analyzed. Survival analysis was conducted using the TCGA breast cancer cohort. Cases with available gene expression and overall survival data were included, and 5-year overall survival was evaluated using the survival package in R software. MYC and EPHB4 expression levels were dichotomized into high- and low-expression groups according to the best cutoff value. Kaplan–Meier survival analysis was then performed to compare overall survival between MYC-high and MYC-low groups, between EPHB4-high and EPHB4-low groups, and among groups defined by combined MYC and EPHB4 expression status.

Expression correlation analysis was performed using the GEO dataset GSE25055. Gene expression values from all available breast cancer samples were used to examine the associations between MYC and EPHB4, and between EPHB4 and PSMB5. Correlation coefficients and corresponding *P* values were calculated using the ggstatsplot R package.

### Statistical analysis

Data are presented as mean ± SEM. Statistical analyses were performed using GraphPad Prism 9 (GraphPad Software, San Diego, CA, USA). For comparisons between two groups, a two-tailed unpaired Student’s t-test was used. For comparisons among multiple groups, one-way ANOVA followed by the appropriate multiple-comparisons test was used. For tumor growth curve analyses, two-way ANOVA was used. The value of *n* indicates the number of independent biological replicates for in vitro experiments or the number of mice for in vivo studies. Sample sizes for animal experiments were chosen based on our previous experience with these xenograft models and on group sizes commonly used in comparable studies. A *p* value < 0.05 was considered statistically significant.

## Supplementary information

CDDIS-26-0393_Supplementary data

uncropped western blots

Table S4

## Data Availability

All the data in this study are available from the corresponding author on reasonable request.
