# Supplementary material for: EphB4 inhibition defines a druggable synthetic-lethal vulnerability in MYC-driven triple-negative breast cancer
Source: Cell Death Dis. 2026 Jun 26;17(1):792. doi: 10.1038/s41419-026-09020-5 (PMC13558723; doi:10.1038/s41419-026-09020-5)

Figure 1C

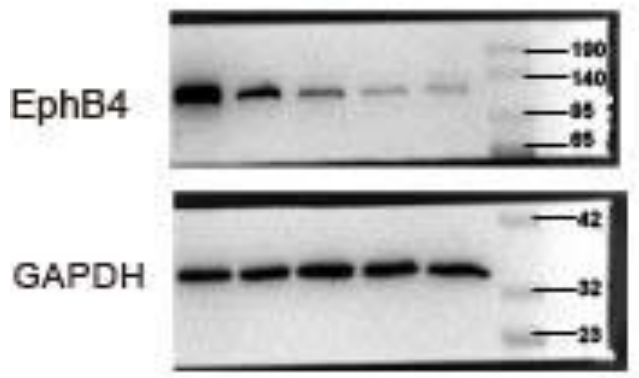

Figure 1E

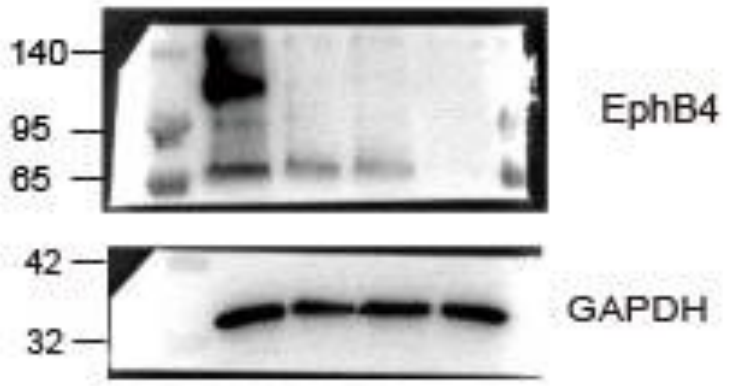

Figure 1F

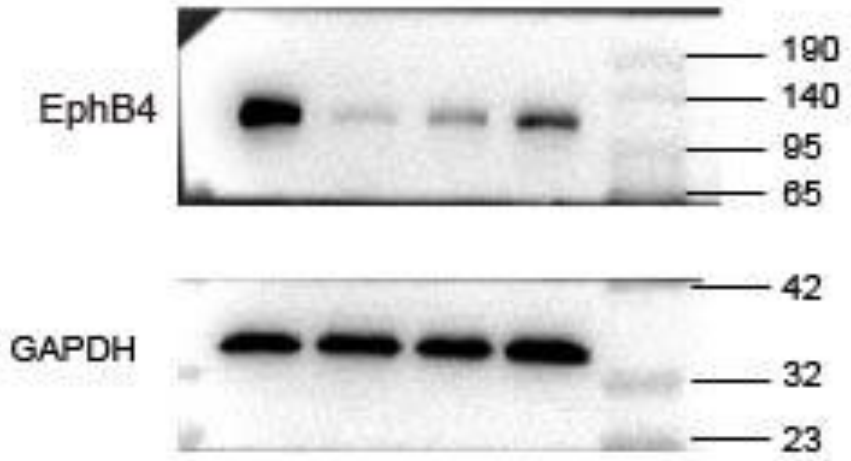

Figure 2B

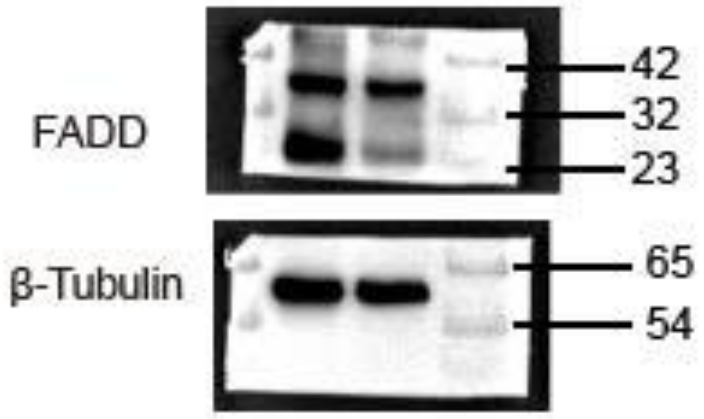

Figure 2C

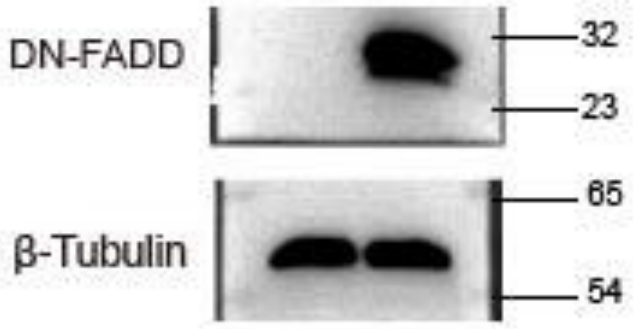

Figure 2F

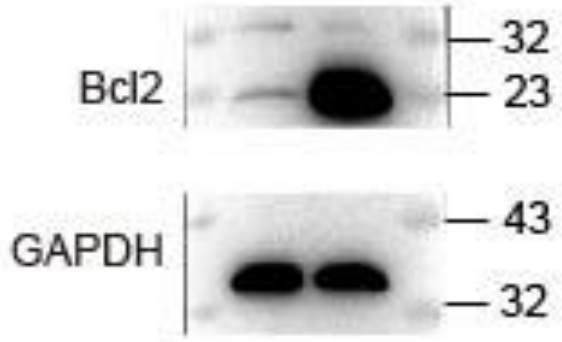

Figure 2G

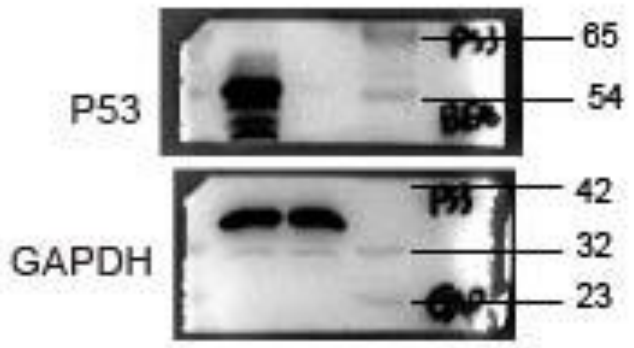

Figure 2K

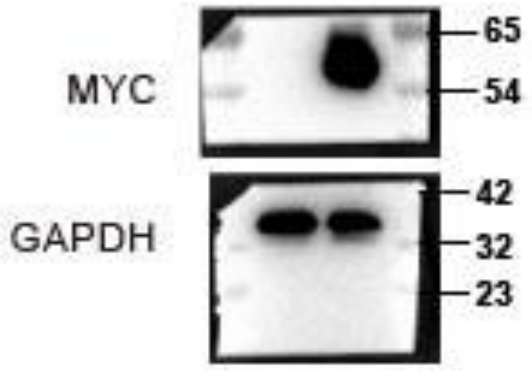

Figure 2L

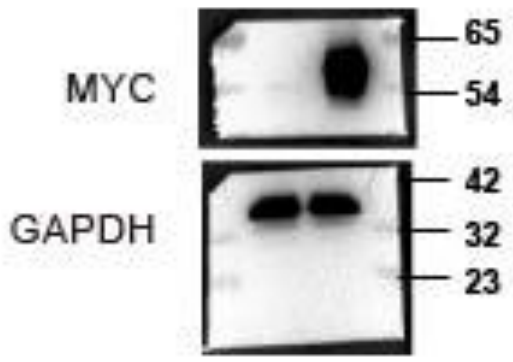

Figure 3A

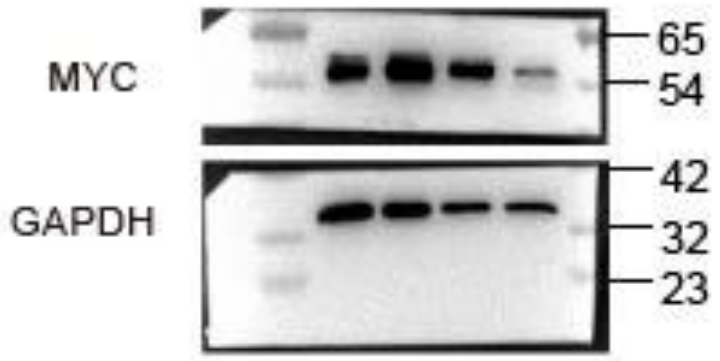

Figure 3B

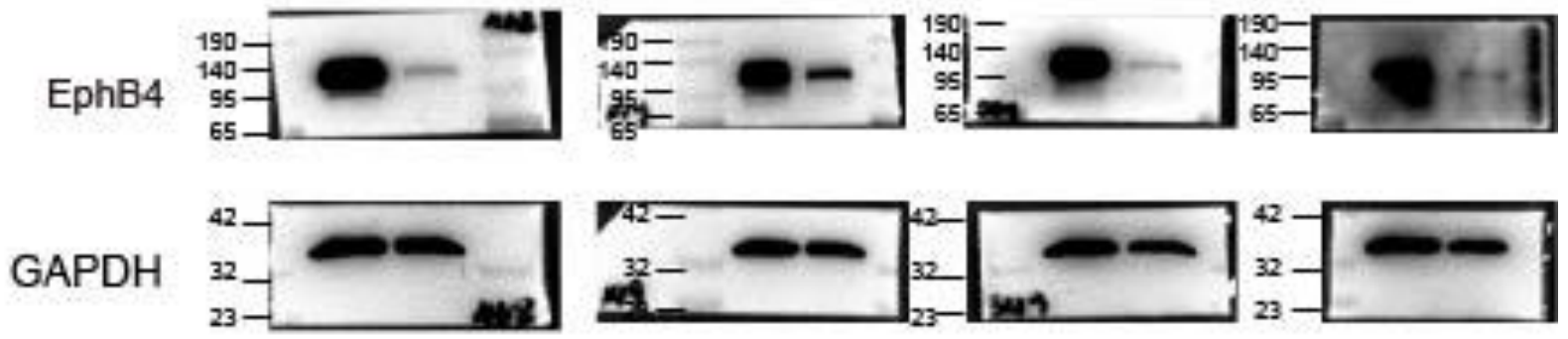

Figure 3E

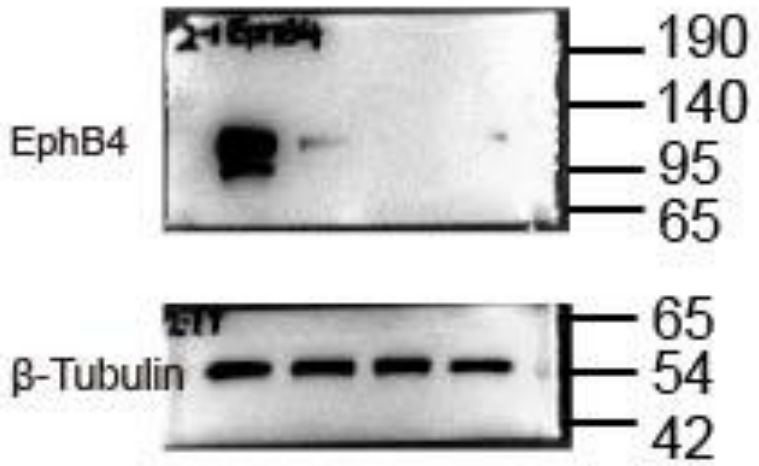

Figure 3F

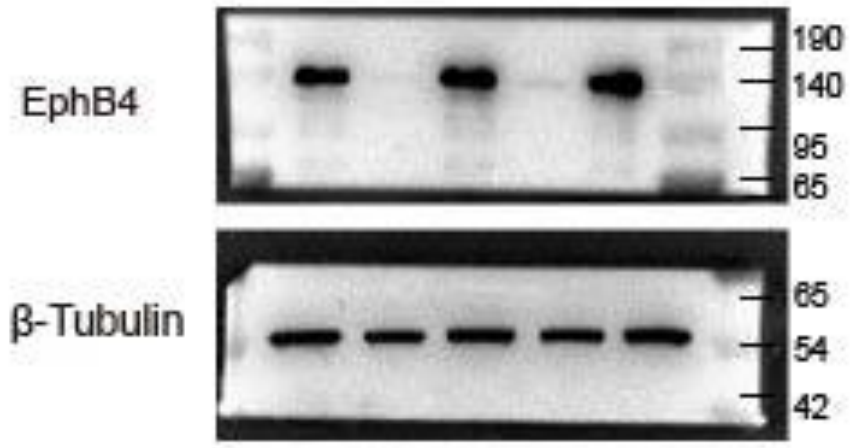

Figure 3H

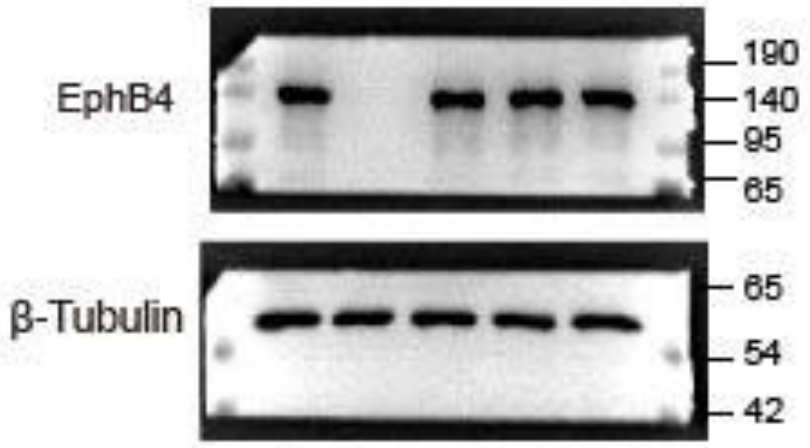

Figure 3M

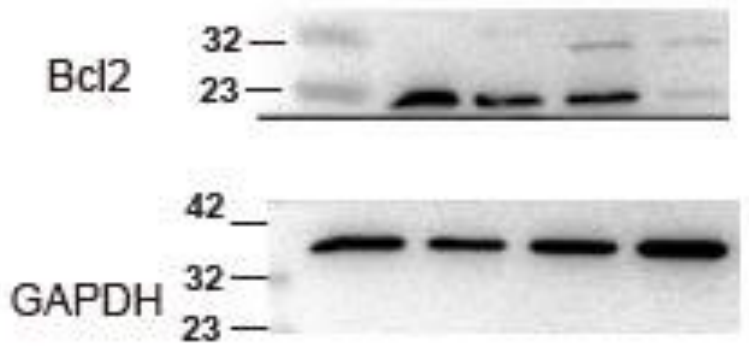

Figure 4F

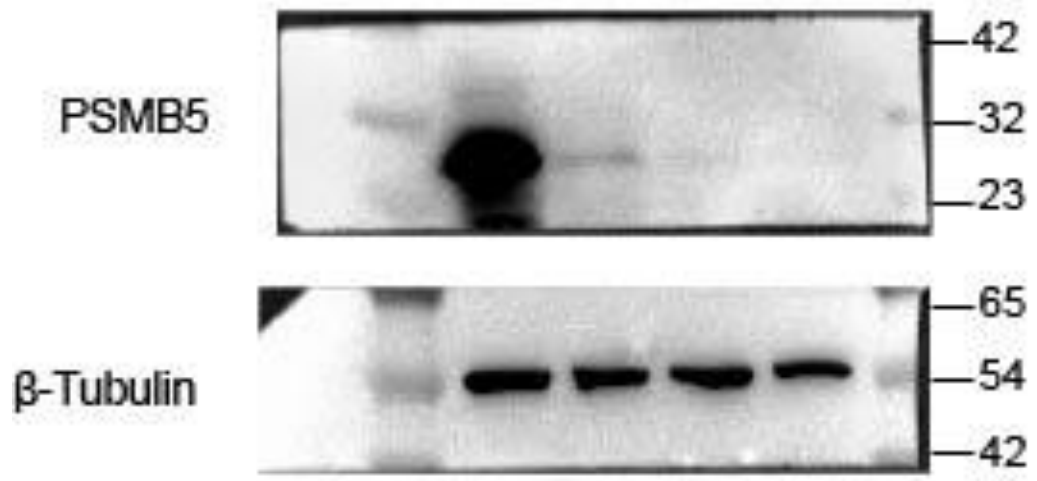

Figure 5B

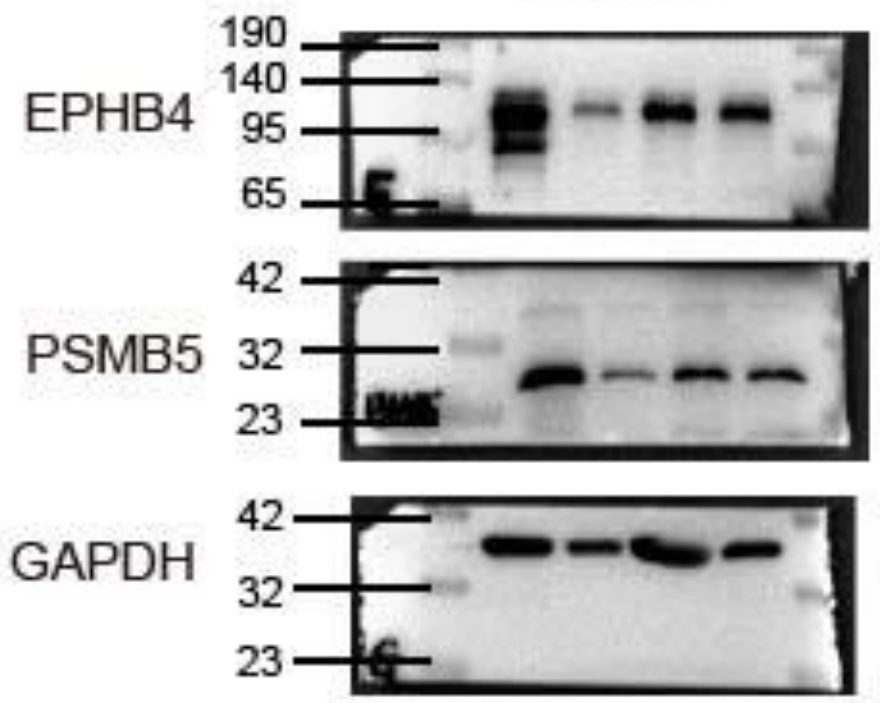

Figure 5D

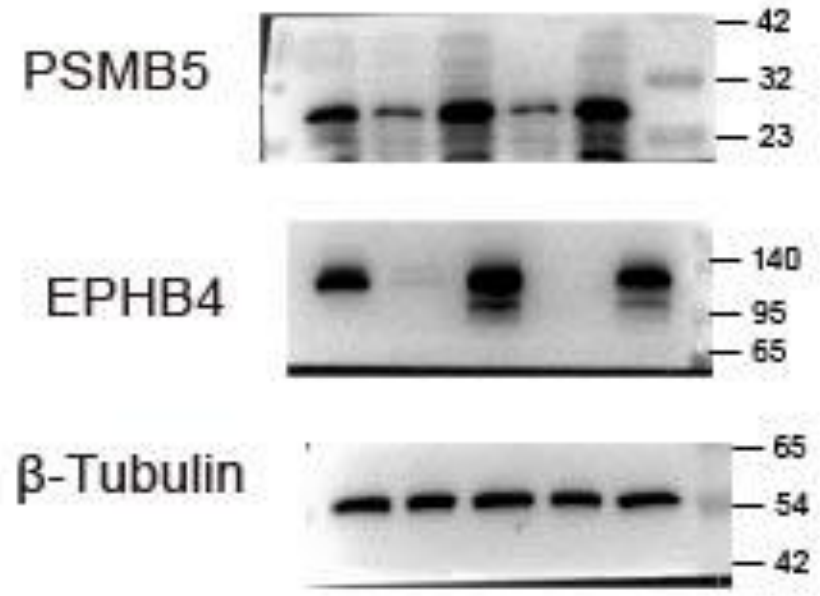

Figure 6C

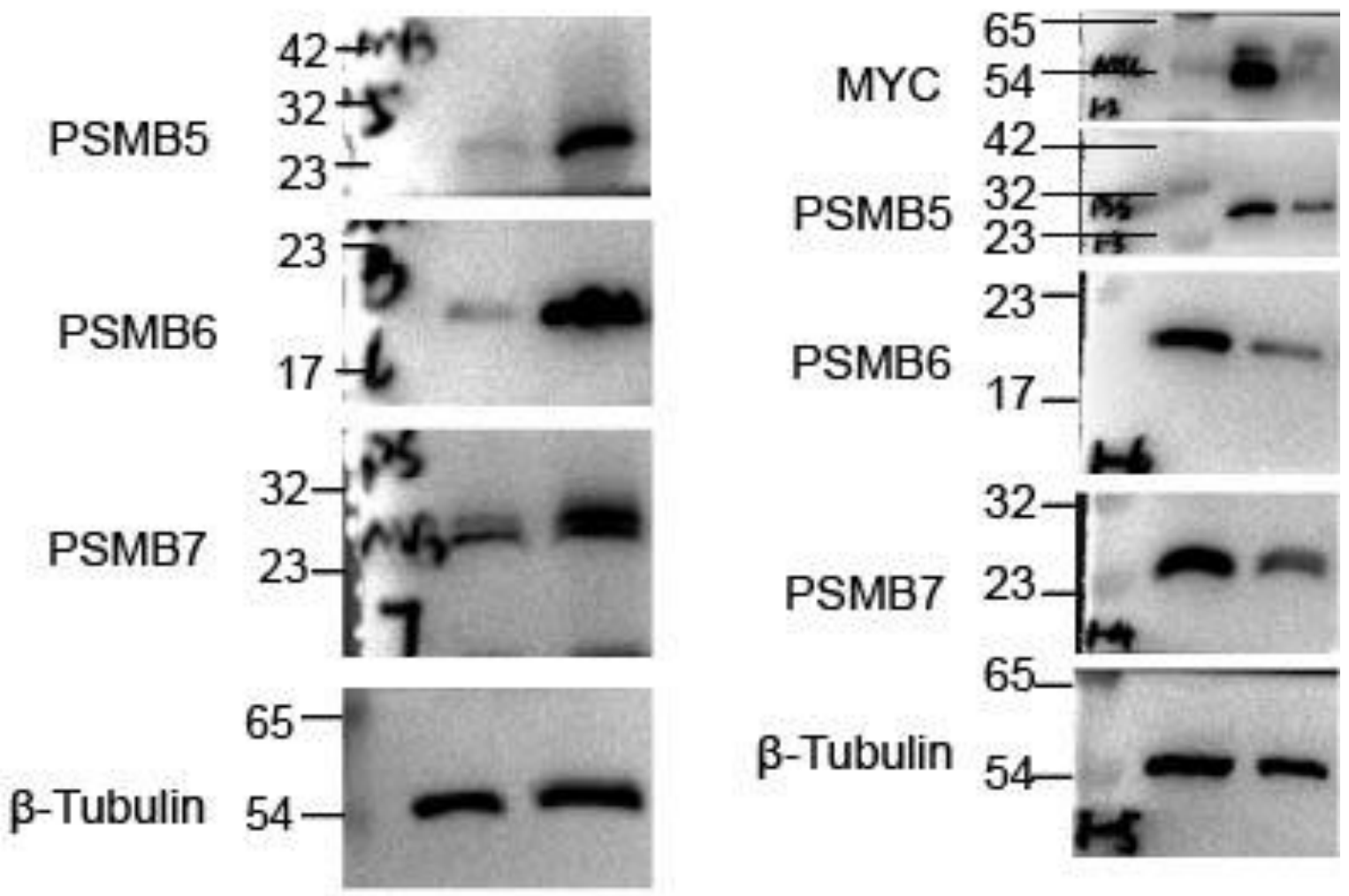

Figure 5F

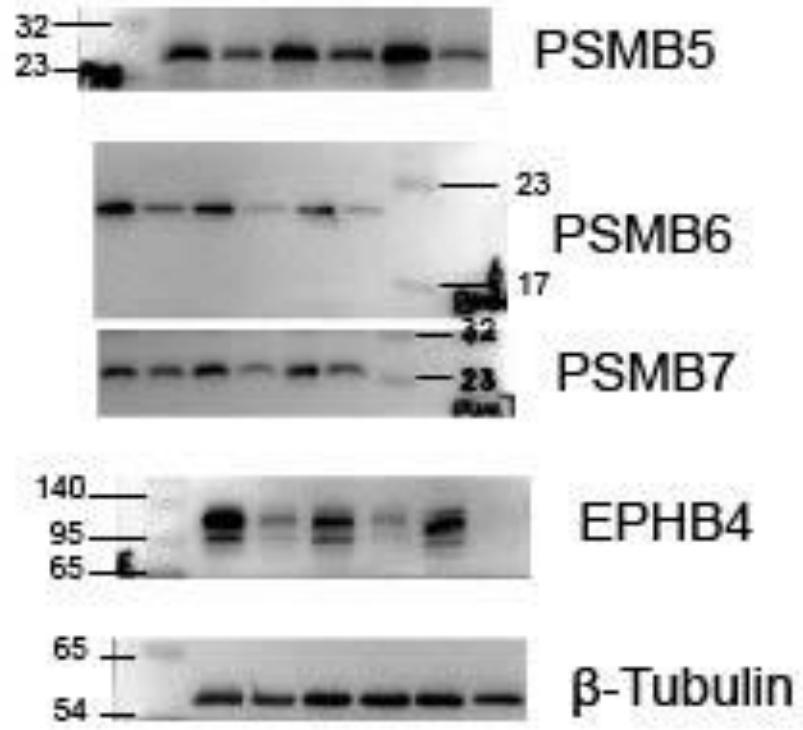

Figure 5J

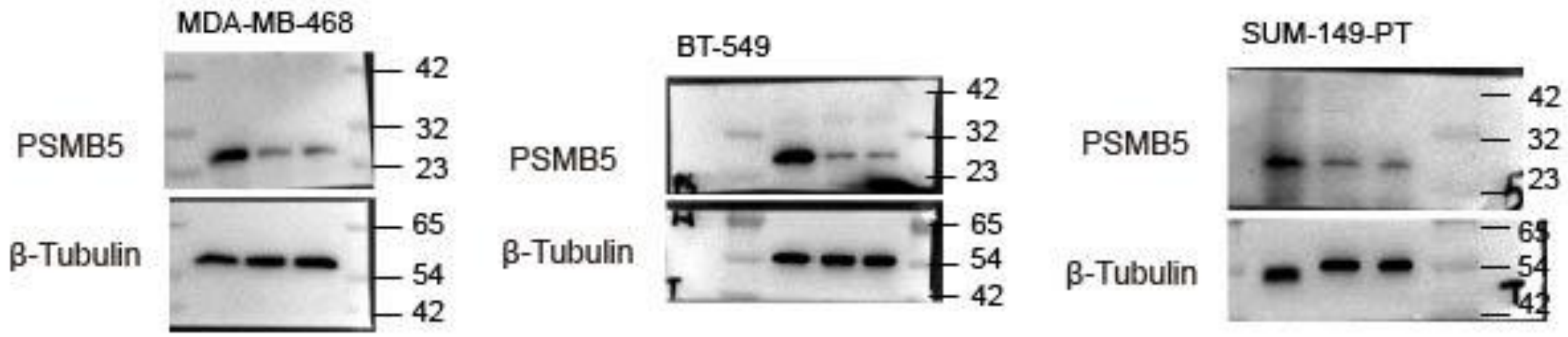

Figure 5L

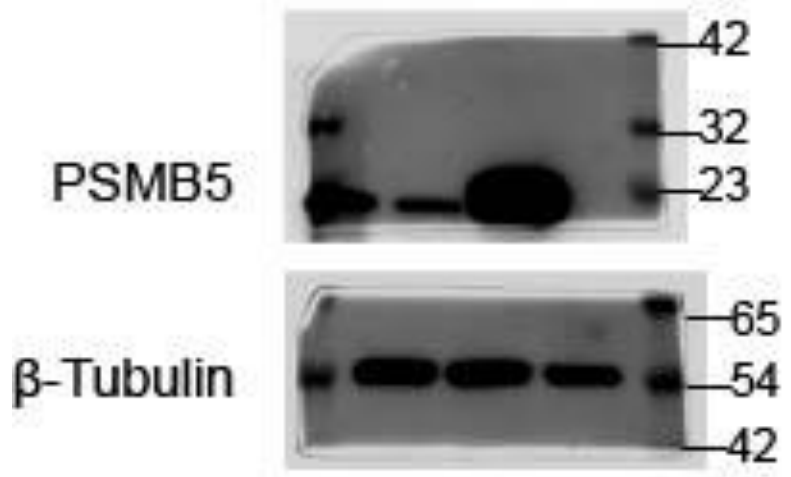

Figure S3B

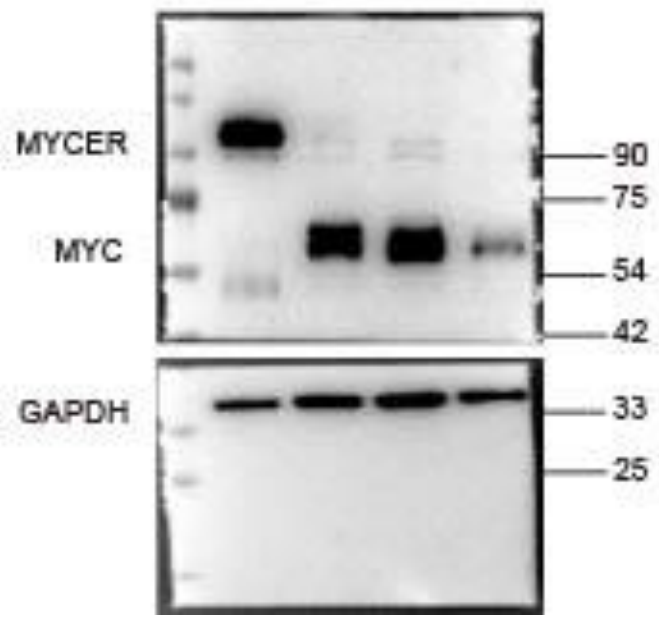

Figure S3K

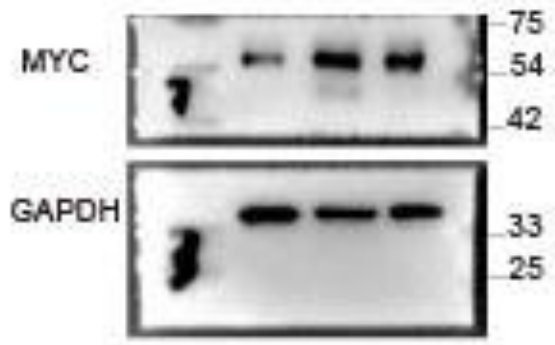

Supplement: Supplementary file 1 [file 41419_2026_9020_MOESM2_ESM.pdf]
