# Supplementary material for: EphB4 inhibition defines a druggable synthetic-lethal vulnerability in MYC-driven triple-negative breast cancer
Source: Cell Death Dis. 2026 Jun 26;17(1):792. doi: 10.1038/s41419-026-09020-5 (PMC13558723; doi:10.1038/s41419-026-09020-5)
Supplement: Supplementary file 2 [file 41419_2026_9020_MOESM1_ESM.docx]

**Figure S1. Related to Figure 1.**

(A-C) Densitometric quantification of EphB4 protein levels corresponding to the immunoblot analyses shown in Fig. 1C, 1E, and 1F. EphB4 protein levels were normalized to GAPDH and expressed relative to the control condition.

(D-F) Apoptosis analysis of U2OS-MYCER (D), HFF-1-MYCER (E), and BEAS-2B-MYCER (F) cells treated with DMSO or the EPHB4 inhibitors NVP-BHG712 (2 μM) or AZ12672857 (1 μM) for 3 days in the presence or absence of OHT.

Graphical data are means ± SEM. *n* = 3. ^***^, *P* < 0.001; ^#^, *P* < 0.0001.

**Figure S2. Related to Figure 2.**

(A-F) Densitometric quantification of immunoblot analyses shown in Fig. 2B (A), Fig. 2C (B), Fig. 2F (C), Fig. 2G (D), Fig. 2K (E), and Fig. 2L (F). Relative protein levels of FADD (A), DN-FADD (B), Bcl-2 (C), p53 (D), and MYC (E, F) were normalized to the corresponding loading control and expressed relative to the control condition.

**Figure S3. Related to Figure 3.**

(A) Densitometric quantification of MYC protein levels corresponding to the immunoblot analysis shown in Fig. 3A. Relative MYC protein levels were normalized to GAPDH and expressed relative to MCF10A cells.

(B-C) Immunoblot analysis comparing MYCER expression in ARPE-19-MYCER cells with endogenous MYC levels in MDA-MB-468, BT-549, and T47D cells, and densitometric quantification of the corresponding immunoblot. GAPDH was used as a loading control. Relative MYC protein levels were normalized to GAPDH and expressed relative to ARPE-19-MYCER cells.

(D) Densitometric quantification of EphB4 protein levels corresponding to the immunoblot analysis shown in Fig. 3B. Relative EphB4 protein levels were normalized to β-tubulin and expressed relative to the respective control conditions.

(E) Apoptosis analysis of T47D-MYCER cells treated with DMSO or NVP-BHG712 (2 μM) in the presence or absence of OHT.

(F) Densitometric quantification of EphB4 protein levels corresponding to the immunoblot analysis shown in Fig. 3E. Relative EphB4 protein levels were normalized to β-tubulin and expressed relative to the shGFP control.

(G) Densitometric quantification of EphB4 protein levels corresponding to the immunoblot analysis shown in Fig. 3F. Relative EphB4 protein levels were normalized to β-tubulin and expressed relative to the shGFP control.

(H) Densitometric quantification of EphB4 protein levels corresponding to the immunoblot analysis shown in Fig. 3H. Relative EphB4 protein levels were normalized to β-tubulin and expressed relative to the shGFP control.

(I) mRNA expression levels of *EFNB2* in BT-549 cells expressing doxycycline-inducible GFP shRNA or three independent *EFNB2* shRNAs (#1, #2, or #3).

(J) Growth kinetics of ARPE-19-STR, MDA-MB-468, and BT-549 cells assessed by CCK-8 assay at the indicated time points.

(K-L) Immunoblot analysis of MYC expression in ARPE-19-STR, MDA-MB-468, and BT-549 cells, and densitometric quantification of the corresponding immunoblot. GAPDH was used as a loading control and expressed relative to ARPE-19-STR cells.

(M) Densitometric quantification of Bcl-2 protein levels corresponding to the immunoblot analysis shown in Fig. 3M. Relative Bcl-2 protein levels were normalized to GAPDH and expressed relative to MCF10A cells.

(N) Apoptosis analysis of MDA-MB-468, SUM-149-PT, and BT-549 cells treated with the EPHB4 inhibitor NVP-BHG712 (2 μM) and/or ABT-199 (6 μM) for 3 days.

(O) Body weight of mice from the experiment shown in Figure 3O was monitored throughout the treatment period.

Graphical data are means ± SEM. *n* = 3. ^*^, *P* < 0.05; ^**^, *P* < 0.01; ^***^, *P* < 0.001; ^****^, *P* < 0.0001.

**Figure S4. Related to Figure 4.**

(A) Relative FPKM values of the six candidate genes identified from the overlap between MYC-upregulated genes and genes downregulated by EPHB4 knockdown in MCF10A-MYCER cells.

(B) Densitometric quantification of PSMB5 protein levels corresponding to the immunoblot analysis shown in Fig. 4F. Relative PSMB5 levels were normalized to β-tubulin and expressed relative to the shGFP control.

**Figure S5. Related to Figure 5.**

(A) Densitometric quantification of EphB4 and PSMB5 protein levels corresponding to the immunoblot analysis shown in Fig. 5B. Relative protein levels were normalized to GAPDH and expressed relative to the shGFP group.

(B) Densitometric quantification of PSMB5 and EphB4 protein levels corresponding to the immunoblot analysis shown in Fig. 5D. Relative protein levels were normalized to β-tubulin and expressed relative to the corresponding empty vector group.

(C) Relative *PSMB5,* *PSMB6,* and *PSMB7* mRNA levels in MDA-MB-468 cells expressing doxycycline-inducible GFP shRNA or three independent *EPHB4* shRNAs (#1, #2, or #3), treated with Dox for 2 days. Bars are mean ± SEM. #, P < 0.0001; significantly different from control cells.

(D) Densitometric quantification of PSMB5, PSMB6, PSMB7, and EphB4 protein levels corresponding to the immunoblot analysis shown in Fig. 5F. Relative protein levels were normalized to β-tubulin and expressed relative to the corresponding control group.

(E) Relative proteasome activities associated with PSMB5, PSMB6, and PSMB7 in MDA-MB-468 cells treated with DMSO, carfilzomib (100 nM), or NVP-BHG712 (2 μM), as measured by fluorogenic substrate hydrolysis assays.

(F) Densitometric quantification of PSMB5 protein levels corresponding to the immunoblot analysis shown in Fig. 5J. Relative protein levels were normalized to β-tubulin and expressed relative to the shGFP group.

(G) Densitometric quantification of PSMB5 protein levels corresponding to the immunoblot analysis shown in Fig. 5L. Relative protein levels were normalized to β-tubulin and expressed relative to the empty vector group.

(H) Apoptosis analysis of MDA-MB-468 cells treated with DMSO or carfilzomib (100 nM) in the presence or absence of MYCi975 (6 μM).

Data are means ± SEM. *n* = 3. ^**^, *P* < 0.01; ^***^, *P* < 0.001; ^#^, *P* < 0.0001.

**Figure S6. Related to Figure 6.**

Analysis of publicly available MYC ChIP-seq data (ChIP-Atlas) in BT-549 cells treated with either DMSO or MYCMI-6.

**Figure S7. Related to Figure 6.**

(A) Volcano plot of differentially expressed proteasome subunit-coding genes (DEGs) in MYC-activated (OHT+) versus control (OHT-) MCF10A-MYCER cells. Each dot represents a DEG, with colored circles indicating significantly upregulated or downregulated genes.

(B-C) Densitometric quantification of immunoblot analyses shown in Fig. 6C. Relative protein levels of PSMB5, PSMB6, and PSMB7 in MCF10A-MYCER cells treated with or without OHT (B).

Relative protein levels of MYC, PSMB5, PSMB6, and PSMB7 in MDA-MB-468 cells expressing control shRNA (shGFP) or sh*MYC* (C). Protein levels were normalized to β-tubulin and expressed relative to the respective control conditions.

(D) Luciferase reporter assay in MCF10A-MYCER cells stably expressing doxycycline-inducible EPHB4 shRNA #2 and the PSMB5 promoter reporter. Cells were treated with or without OHT and doxycycline as indicated. Luciferase activity was normalized to Renilla.

(E-G) Kaplan–Meier analysis of 5-year overall survival in the TCGA breast cancer cohort according to MYC expression (E), EPHB4 expression (F), or combined MYC and EPHB4 expression status (G).

(H) Correlation analysis of MYC and EPHB4 expression (left) and of EPHB4 and PSMB5 expression (right) in the GEO breast cancer dataset GSE25055.

For panels B-D, data are mean ± SEM, n = 3. n.s., not significant; #, P < 0.0001
